# Supplementary material for: Biomass removal promotes plant diversity after short-term de-intensification of managed grasslands
Source: PLoS One. 2023 Jun 29;18(6):e0287039. doi: 10.1371/journal.pone.0287039 (PMC10310043; doi:10.1371/journal.pone.0287039)
Supplement: S1 Fig — In all three regions, four grasslands in the Schorfheide-Chorin, six grasslands in the Schwäbische Alb, and 6 grasslands in the Hainich-Dün (16 in total) were selected, where background land-use varied in mowing frequency, grazing intensity and fertilizer input. Within those grasslands, we established four 7×7 m treatments as a full factorial design with: fertilized & biomass removal, fertilized & reduced biomass removal (grazing stopped, but one late cut), unfertilized & biomass removal, unfertilized & reduced biomass removal. In all plots a 1×1 m subplot was established, where we measured plant biomass and performed vegetation surveys. Plot and subplot location was partly randomized in the grassland. (DOCX) [file pone.0287039.s001.docx]

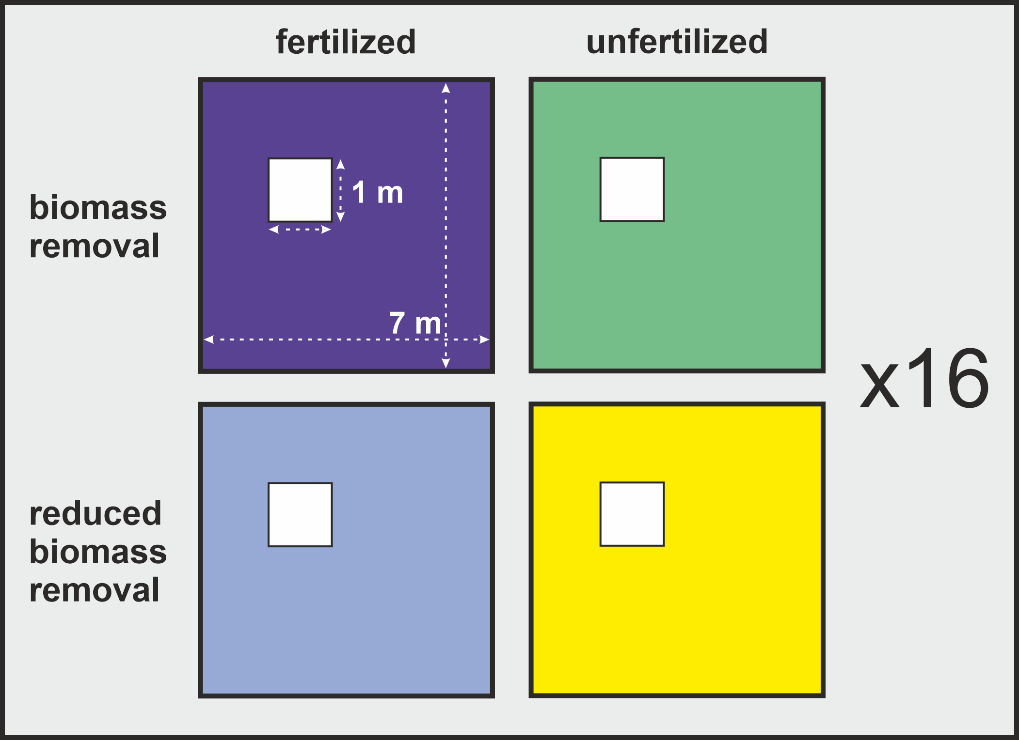


**S1 Figure: Overview showing the full factorial design.** In all three regions, four fields in the Schorfheide-Chorin, six fields in the Schwäbische Alb, and 6 fields in the Hainich-Dün (16 in total) were selected, where background land-use varied in mowing frequency, grazing intensity and fertilizer input. Within those fields, we established four 7×7 m treatments as a full factorial design with: fertilized & biomass removal, fertilized & reduced biomass removal (grazing stopped, but one late cut), unfertilized & biomass removal, unfertilized & reduced biomass removal. In all plots a 1×1 m subplot was established, where we measured plant biomass and performed vegetation surveys. Plot and subplot location was partly randomized in the field.
