# Supplementary material for: Biomass removal promotes plant diversity after short-term de-intensification of managed grasslands
Source: PLoS One. 2023 Jun 29;18(6):e0287039. doi: 10.1371/journal.pone.0287039 (PMC10310043; doi:10.1371/journal.pone.0287039)
Supplement: S2 Fig — Response of species richness (m-2) on the fertilized & biomass removal (+F+R), unfertilized & biomass removal (-F+R), unfertilized & reduced biomass removal (-F-R) and fertilized & reduced biomass removal (+F-R) treatment for all three regions (Alb: Schwäbische Alb; Sch: Schorfheide-Chorin; Hai: Hainich-Dün). Whiskers correspond to the first and third quartiles. Treatments are colour coded as fertilization & biomass removal: purple; fertilized & reduced biomass removal: bright blue, unfertilized & biomass removal: green; unfertilized & reduced biomass removal: yellow. We did not observe any clear significant differences between any of the treatments (S2 Table). (DOCX) [file pone.0287039.s002.docx]

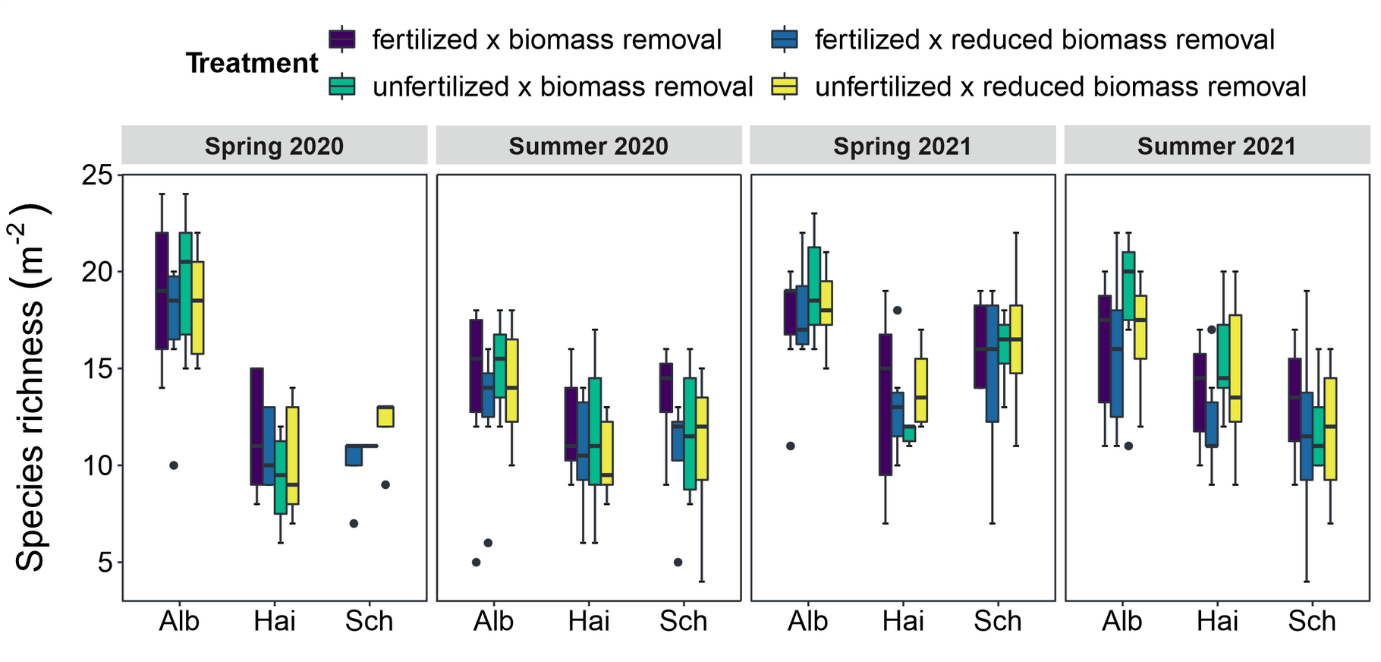


**S2 Figure: Species richness across treatments.** Response of species richness (m-2) on the fertilized & biomass removal (+F+R), unfertilized & biomass removal (-F+R), unfertilized & reduced biomass removal (-F-R) and fertilized & reduced biomass removal (+F-R) treatment for all three regions (Alb: Schwäbische Alb; Sch: Schorfheide-Chorin; Hai: Hainich-Dün). Whiskers correspond to the first and third quartiles. Treatments are colour coded as fertilization & biomass removal: purple; fertilized & reduced biomass removal: bright blue, unfertilized & biomass removal: green; unfertilized & reduced biomass removal: yellow. We did not observe any clear significant differences between any of the treatments (S2 Table).
