# Supplementary material for: Biomass removal promotes plant diversity after short-term de-intensification of managed grasslands
Source: PLoS One. 2023 Jun 29;18(6):e0287039. doi: 10.1371/journal.pone.0287039 (PMC10310043; doi:10.1371/journal.pone.0287039)
Supplement: S3 Fig — Response of Shannon diversity (m-2) on the fertilized & biomass removal (+F+R), unfertilized & biomass removal (-F+R), unfertilized & reduced biomass removal (-F-R), and fertilized & reduced biomass removal (+F-R) treatment for all three regions (Alb: Schwäbische Alb; Sch: Schorfheide-Chorin; Hai: Hainich-Dün). Whiskers correspond to the first and third quartiles. Treatments are colour coded as fertilization & biomass removal: purple; fertilized & reduced biomass removal: bright blue, unfertilized & biomass removal: green; unfertilized & reduced biomass removal: yellow. Bars sharing a letter (a,b) do not differ significantly (p<0.05, S10 Table). We only detected significant differences between treatments in summer 2020 within the Schwäbische Alb, thus no letters are shown for other years, seasons or regions. (DOCX) [file pone.0287039.s003.docx]

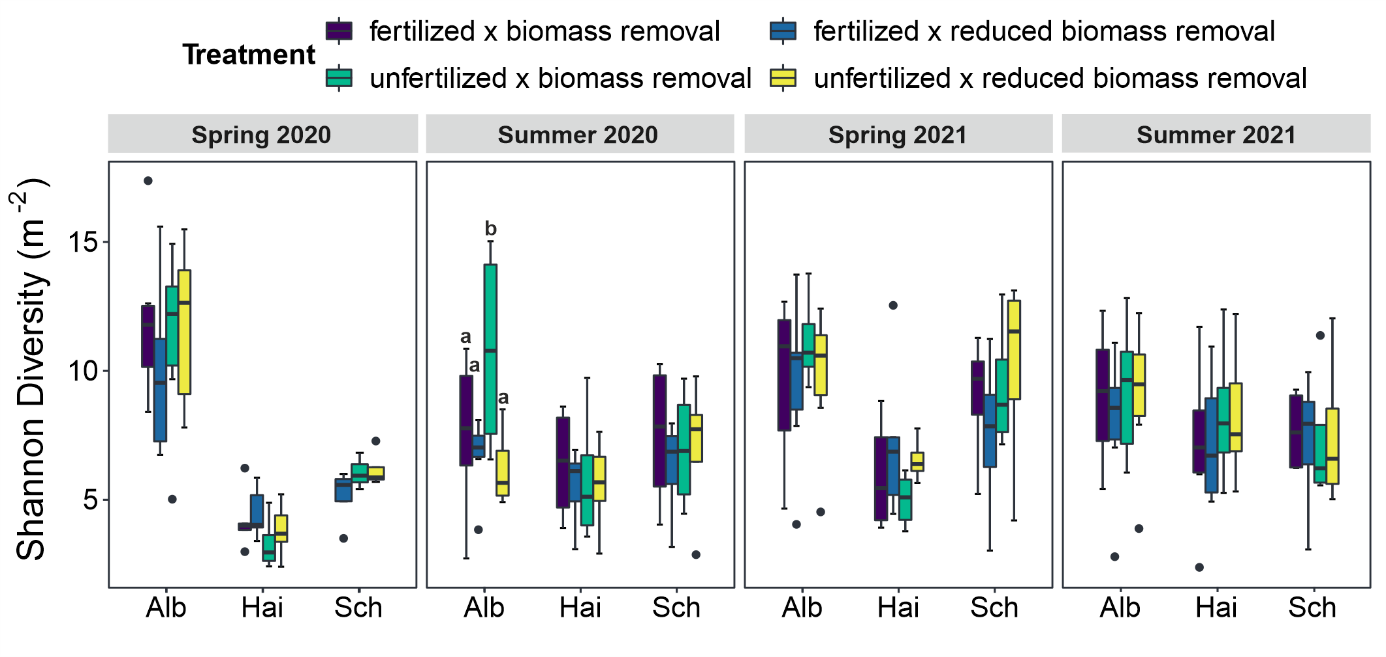


**S3 Figure:** **Shannon diversity per treatment.** Response of Shannon diversity (m^-2^) on the fertilized & biomass removal (+F+R), unfertilized & biomass removal (-F+R), unfertilized & reduced biomass removal (-F-R), and fertilized & reduced biomass removal (+F-R) treatment for all three regions (Alb: Schwäbische Alb; Sch: Schorfheide-Chorin; Hai: Hainich-Dün). Whiskers correspond to the first and third quartiles. Treatments are colour coded as fertilization & biomass removal: purple; fertilized & reduced biomass removal: bright blue, unfertilized & biomass removal: green; unfertilized & reduced biomass removal: yellow. Bars sharing a letter (a,b) do not differ significantly (p<0.05, S10 Table). We only detected significant differences between treatments in summer 2020 within the Schwäbische Alb, thus no letters are shown for other years, seasons or regions.
