# Supplementary material for: Biomass removal promotes plant diversity after short-term de-intensification of managed grasslands
Source: PLoS One. 2023 Jun 29;18(6):e0287039. doi: 10.1371/journal.pone.0287039 (PMC10310043; doi:10.1371/journal.pone.0287039)
Supplement: S6 Fig — Correlation between background LUI Index components, background fertilization intensity (kg N m-3 year-1), background mowing intensity (cuts year-1) and background grazing intensity (Livestock units * d ha-1 year-1) averaged across the years 2017–2019 and combined for all regions. (DOCX) [file pone.0287039.s006.docx]

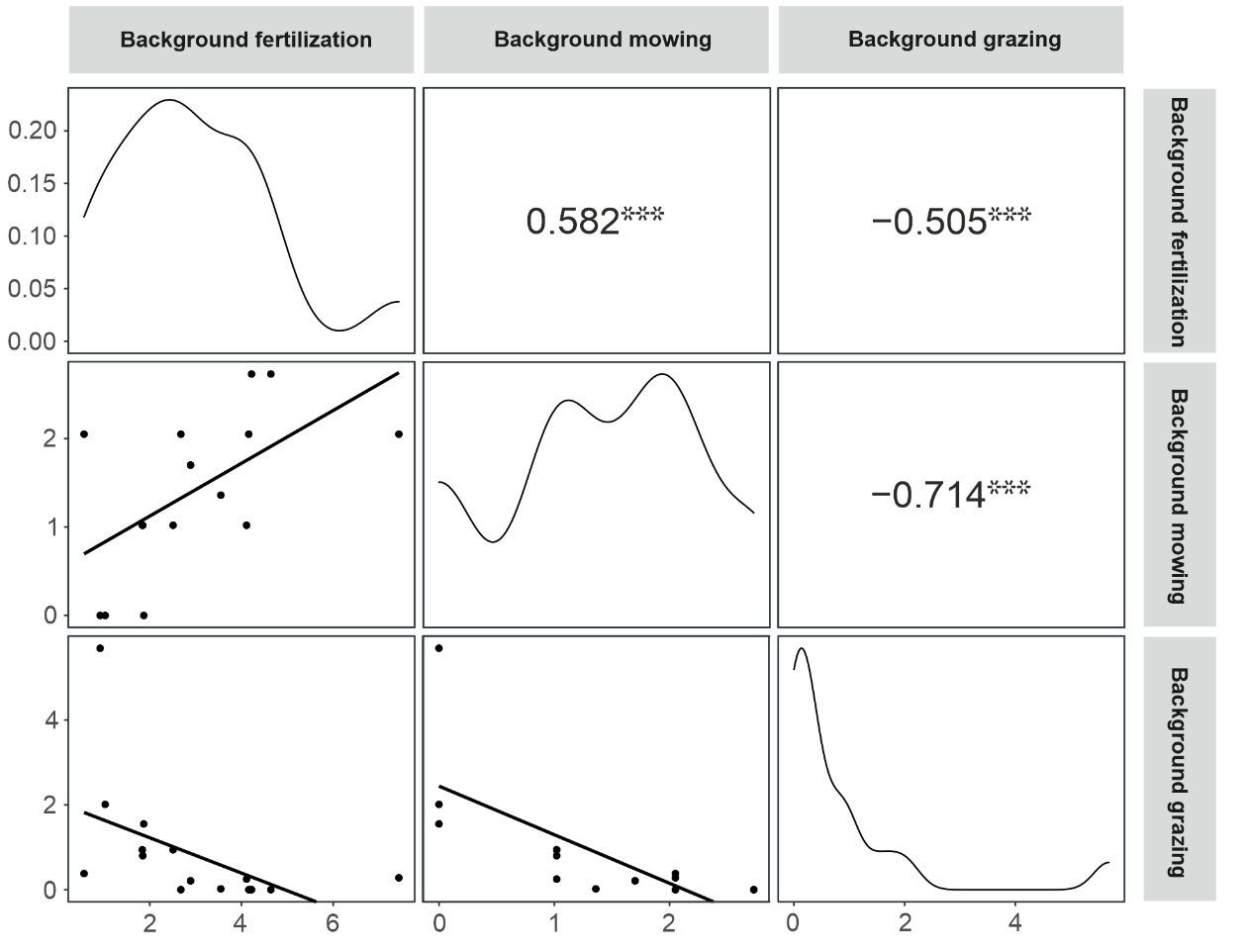


**S6 Figure:** **Correlation between land-use components.** Correlation between background LUI Index components, background fertilization intensity (kg N m-3 year-1), background mowing intensity (cuts year-1) and background grazing intensity (Livestock units * d ha-1 year-1) averaged across the years 2017-2019 and combined for all regions.
