# Supplementary material for: Biomass removal promotes plant diversity after short-term de-intensification of managed grasslands
Source: PLoS One. 2023 Jun 29;18(6):e0287039. doi: 10.1371/journal.pone.0287039 (PMC10310043; doi:10.1371/journal.pone.0287039)
Supplement: S8 Fig — Correlation matrix plot for the relationships between richness and Shannon diversity as well as the response variables standing biomass, biomass production, light availability, and background fertilization intensity for summer of both 2020 and 2021, and colour coded for each region separately (Alb: Schwäbische Alb (red); Sch: Schorfheide-Chorin (blue); Hai: Hainich-Dün (green)). (DOCX) [file pone.0287039.s008.docx]

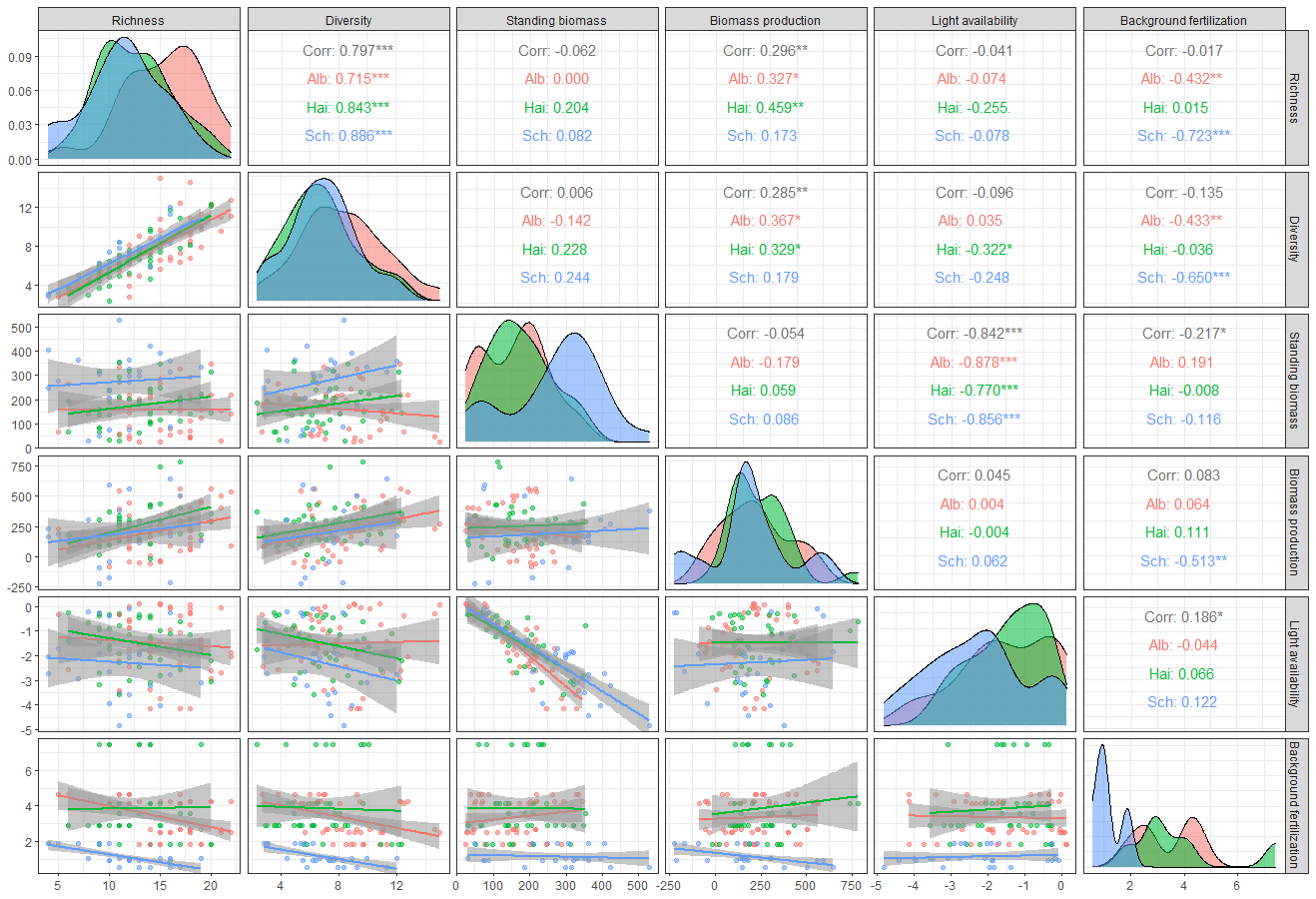


**S8 Figure:** **Correlation between variables of SEM in summer.** Correlation matrix plot for the relationships between richness and Shannon diversity as well as the response variables standing biomass, biomass production, light availability, and background fertilization intensity for summer of both 2020 and 2021, and colour coded for each region separately (Alb: Schwäbische Alb (red); Sch: Schorfheide-Chorin (blue); Hai: Hainich-Dün (green)).
