# Supplementary material for: Biomass removal promotes plant diversity after short-term de-intensification of managed grasslands
Source: PLoS One. 2023 Jun 29;18(6):e0287039. doi: 10.1371/journal.pone.0287039 (PMC10310043; doi:10.1371/journal.pone.0287039)
Supplement: S9 Fig — Partial residuals were extracted from model predicting species richness or Shannon diversity of respective SEM model. For more details on model estimates and significance see S3–S6 Tables. (DOCX) [file pone.0287039.s009.docx]

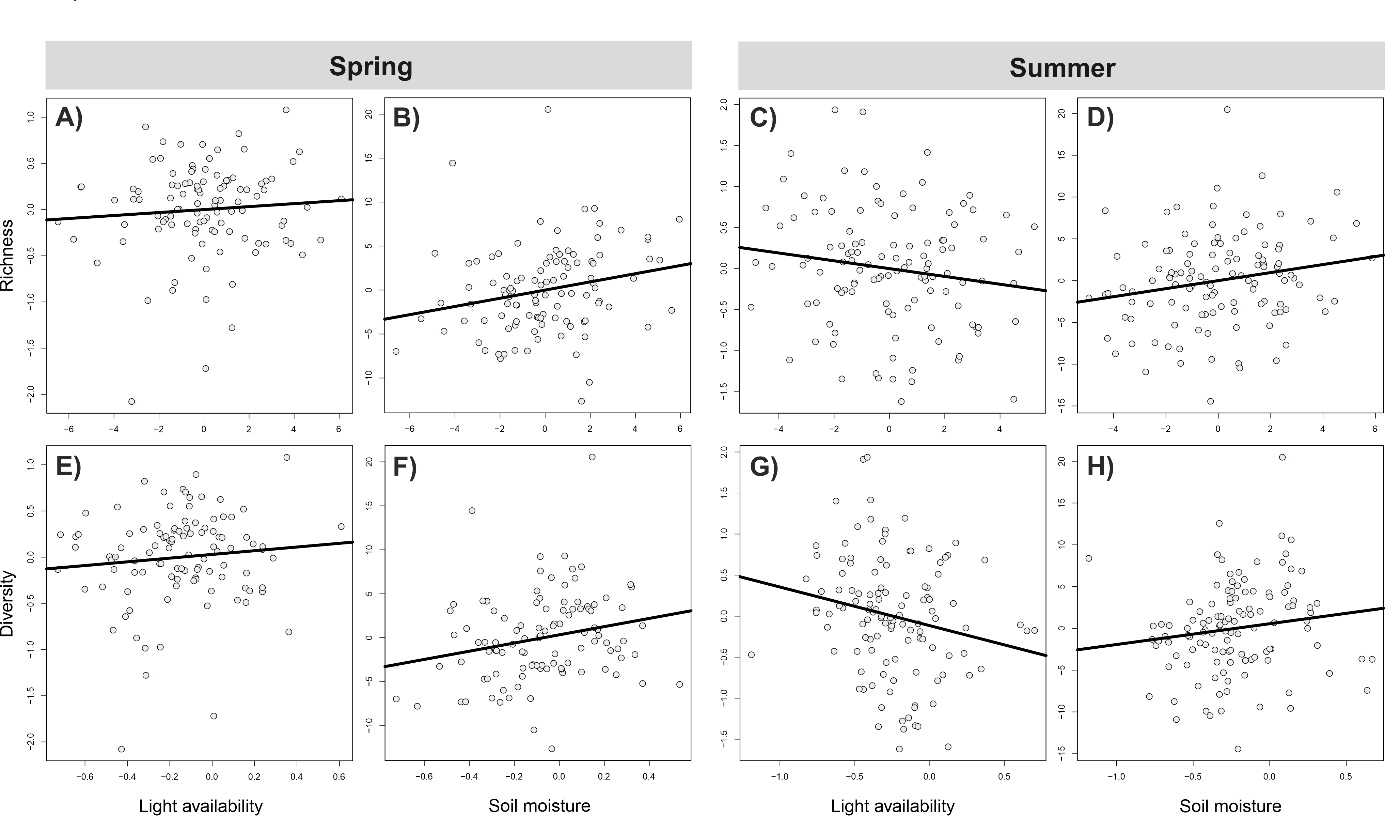


**S9 Figure:** **Partial residual plots**, Partial residual plots showing the effect of light availability and soil moisture on richness in spring (A,B) and summer (C,D), as well as on Shannon diversity in spring (E,F) and summer (G,H). Partial residuals were extracted from model predicting species richness or Shannon diversity of respective SEM model. For more details on model estimates and significance see S3 - S6 Tables.
