# Supplementary material for: Biomass removal promotes plant diversity after short-term de-intensification of managed grasslands
Source: PLoS One. 2023 Jun 29;18(6):e0287039. doi: 10.1371/journal.pone.0287039 (PMC10310043; doi:10.1371/journal.pone.0287039)
Supplement: S10 Fig — Non-metric multidimensional scaling (NMDS) based on Bray–Curtis similarity index for the plant communities for each treatment combination in spring (A-C) and summer (D-F) 2020 (A,D) Schwäbische Alb, (B,E) Hainich-Dün, (C,F) Schorfheide Chorin. Hull volumes represent clusters of plant communities within a given treatment. Treatments are colour coded as fertilized & biomass removal (+F+R): purple; fertilized & reduced biomass removal (+F-R): bright blue, unfertilized & biomass removal (-F+R): green; unfertilized & reduced biomass removal (-F-R): yellow. Detailed model summaries are shown in the Supporting Information (S15 Table). (DOCX) [file pone.0287039.s010.docx]

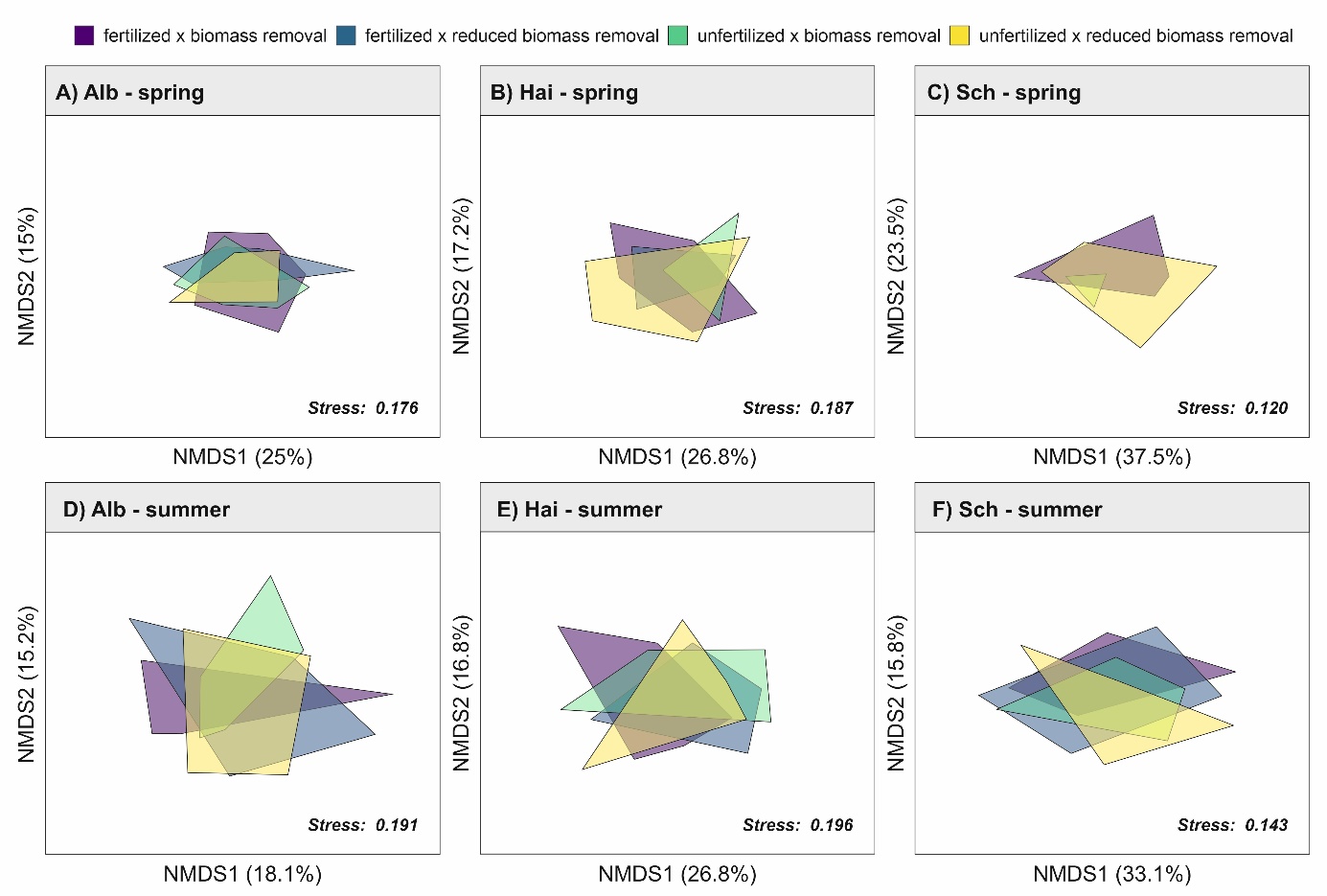


**S10 Figure:** **Changes in community composition in 2020.** Non-metric multidimensional scaling (NMDS) based on Bray–Curtis similarity index for the plant communities for each treatment combination in spring (A-C) and summer (D-F) 2020 (A,D) Schwäbische Alb, (B,E) Hainich-Dün, (C,F) Schorfheide Chorin. Hull volumes represent clusters of plant communities within a given treatment. Treatments are colour coded as fertilized & biomass removal (+F+R): purple; fertilized & reduced biomass removal (+F-R): bright blue, unfertilized & biomass removal (-F+R): green; unfertilized & reduced biomass removal (-F-R): yellow. Detailed model summaries are shown in the Supporting Information (S15 Table).
