# Supplementary material for: Biomass removal promotes plant diversity after short-term de-intensification of managed grasslands
Source: PLoS One. 2023 Jun 29;18(6):e0287039. doi: 10.1371/journal.pone.0287039 (PMC10310043; doi:10.1371/journal.pone.0287039)
Supplement: S11 Fig — Non-metric multidimensional scaling (NMDS) based on Bray–Curtis similarity index for the plant communities for each treatment combination and all regions combined in spring (A) and summer (B) for 2020. R2-values are shown for each axis. Arrows printed in grey represent environmental factors: standing biomass, light availability and soil moisture, while arrows printed in red represent species richness and Shannon diversity. Species names represent the most extreme species according to the NMDS axes. Treatments are colour coded as fertilized & biomass removal (+F+R): purple; fertilized & reduced biomass removal (+F-R): bright blue, unfertilized & biomass removal (-F+R): green; unfertilized & reduced biomass removal (-F-R): yellow. Detailed model summaries are shown in the Supporting Information (S16 Table). (DOCX) [file pone.0287039.s011.docx]

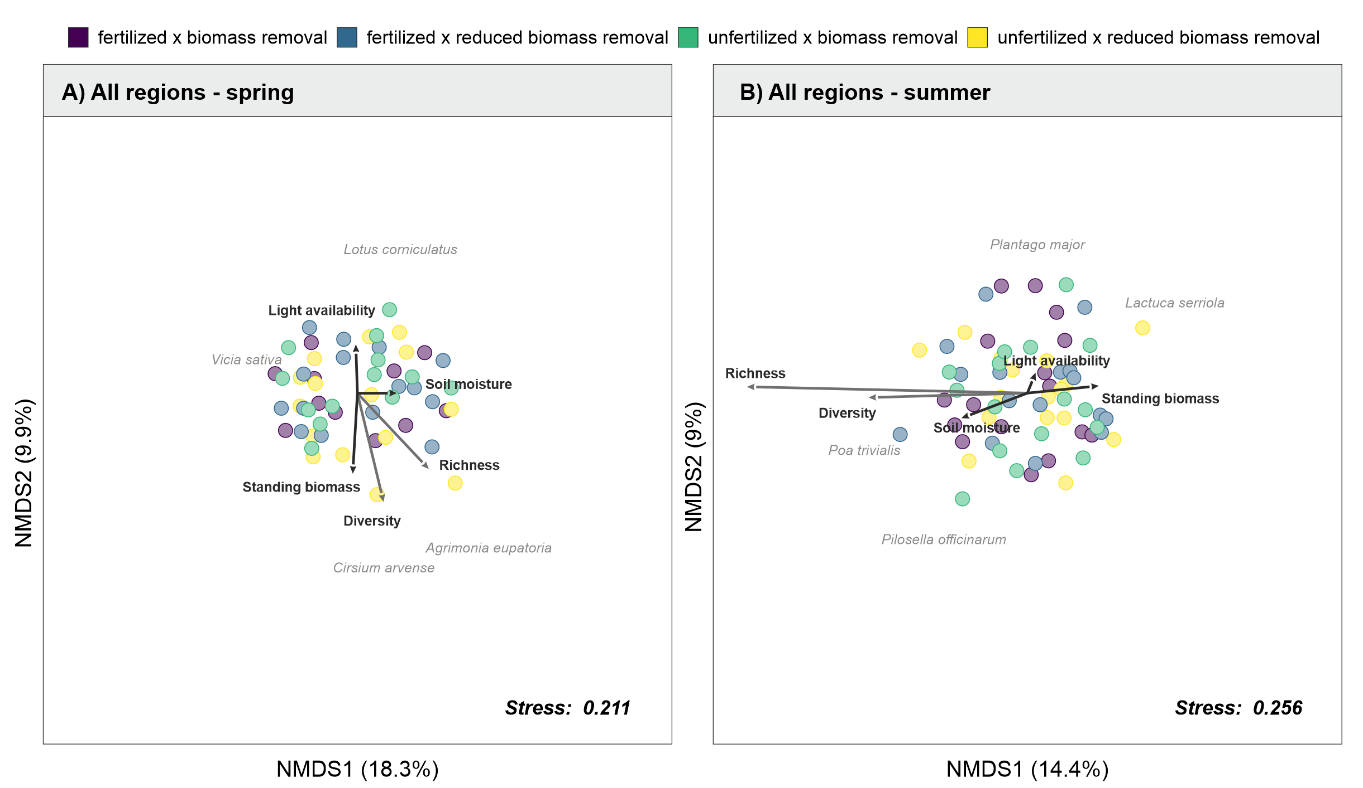


**S11 Figure:** **Community composition and environmental factors in 2020.** Non-metric multidimensional scaling (NMDS) based on Bray–Curtis similarity index for the plant communities for each treatment combination and all regions combined in spring (A) and summer (B) for 2020. R2-values are shown for each axis. Arrows printed in grey represent environmental factors: standing biomass, light availability and soil moisture, while arrows printed in red represent species richness and Shannon diversity. Species names represent the most extreme species according to the NMDS axes. Treatments are colour coded as fertilized & biomass removal (+F+R): purple; fertilized & reduced biomass removal (+F-R): bright blue, unfertilized & biomass removal (-F+R): green; unfertilized & reduced biomass removal (-F-R): yellow. Detailed model summaries are shown in the Supporting Information (S16 Table).
