# Supplementary material for: Biomass removal promotes plant diversity after short-term de-intensification of managed grasslands
Source: PLoS One. 2023 Jun 29;18(6):e0287039. doi: 10.1371/journal.pone.0287039 (PMC10310043; doi:10.1371/journal.pone.0287039)
Supplement: S1 Table — Linear mixed effect model showing the effect of the unfertilized & reduced biomass removal (-F-R), fertilized & reduced biomass removal (+F-R), unfertilized & biomass removal (-F+R) on species richness in comparison with the fertilized & biomass removal treatment for each regions (Alb: Schwäbische Alb; Sch: Schorfheide-Chorin; Hai: Hainich-Dün), as well as for different years and seasons. (DOCX) [file pone.0287039.s012.docx]

**S1 Table:** **Species richness in response to treatments.** Linear mixed effect model showing the effect of the unfertilized & reduced biomass removal (-F-R), fertilized & reduced biomass removal (+F-R), unfertilized & biomass removal (-F+R) on species richness in comparison with the fertilized & biomass removal treatment for each regions (Alb: Schwäbische Alb; Sch: Schorfheide-Chorin; Hai: Hainich-Dün), as well as for different years and seasons. In spring 2020, the interaction between the treatment and region was not part of the most parsimonious model. Note that rows are relative to the intercept.

| **Season** | **Predictor** | **Estimate** | **SE** | **95% CI** | **p value** |
| --- | --- | --- | --- | --- | --- |
| Spring 2020 | Intercept (Alb) | 19.23 | 1.11 | 2.18 | < 0.001 |
|  | Hai | -7.90 | 1.34 | 2.63 | < 0.001 |
|  | Sch | -7.22 | 1.50 | 2.94 | < 0.001 |
|  | -F-R | -0.74 | 1.00 | 1.96 | 0.47 |
|  | +F-R | -1.54 | 1.00 | 1.96 | 0.13 |
|  | -F+R | -0.47 | 1.03 | 2.02 | 0.65 |
| Summer 2020 | Intercept (Alb) | 14.00 | 1.43 | 2.80 | < 0.001 |
|  | Hai | -2.00 | 2.03 | 3.98 | 0.33 |
|  | Sch | -0.50 | 2.27 | 4.45 | 0.83 |
|  | -F-R | 0.17 | 1.32 | 2.59 | 0.90 |
|  | +F-R | -1.17 | 1.32 | 2.59 | 0.38 |
|  | -F+R | 1.17 | 1.32 | 2.59 | 0.38 |
|  | -F-R : Hai | -1.83 | 1.87 | 3.67 | 0.33 |
|  | +F-R : Hai | -0.17 | 1.87 | 3.67 | 0.93 |
|  | -F+R : Hai | -1.67 | 1.87 | 3.67 | 0.38 |
|  | -F-R : Sch | -2.92 | 2.09 | 4.10 | 0.17 |
|  | +F-R : Sch | -1.83 | 2.09 | 4.10 | 0.39 |
|  | -F+R : Sch | -2.92 | 2.09 | 4.10 | 0.17 |
| Spring 2021 | Intercept (Alb) | 17.33 | 1.29 | 2.53 | < 0.001 |
|  | Hai | -3.83 | 1.82 | 3.57 | 0.04 |
|  | Sch | -1.08 | 2.04 | 4.00 | 0.60 |
|  | -F-R | 0.83 | 1.45 | 2.84 | 0.57 |
|  | +F-R | 0.67 | 1.45 | 2.84 | 0.65 |
|  | -F+R | 1.83 | 1.45 | 2.84 | 0.21 |
|  | -F-R : Hai | -0.33 | 2.05 | 4.02 | 0.87 |
|  | +F-R : Hai | -1.00 | 2.05 | 4.02 | 0.63 |
|  | -F+R : Hai | -3.67 | 2.05 | 4.02 | 0.08 |
|  | -F-R : Sch | -0.58 | 2.29 | 4.49 | 0.80 |
|  | +F-R : Sch | -2.42 | 2.29 | 4.49 | 0.30 |
|  | -F+R : Sch | -2.08 | 2.29 | 4.49 | 0.37 |
| Summer 2021 | Intercept (Alb) | 16.17 | 1.52 | 2.98 | < 0.001 |
|  | Hai | -2.33 | 2.15 | 4.21 | 0.29 |
|  | Sch | -2.92 | 2.40 | 4.70 | 0.24 |
|  | -F-R | 0.67 | 1.39 | 2.72 | 0.63 |
|  | +F-R | -0.33 | 1.39 | 2.72 | 0.81 |
|  | -F+R | 2.33 | 1.39 | 2.72 | 0.10 |
|  | -F-R : Hai | 0.00 | 1.97 | 3.86 | 1.00 |
|  | +F-R : Hai | -1.33 | 1.97 | 3.86 | 0.50 |
|  | -F+R : Hai | -0.67 | 1.97 | 3.86 | 0.74 |
|  | -F-R : Sch | -2.17 | 2.20 | 4.31 | 0.33 |
|  | +F-R : Sch | -1.42 | 2.20 | 4.31 | 0.52 |
|  | -F+R : Sch | -3.58 | 2.20 | 4.31 | 0.11 |
