# Supplementary material for: Biomass removal promotes plant diversity after short-term de-intensification of managed grasslands
Source: PLoS One. 2023 Jun 29;18(6):e0287039. doi: 10.1371/journal.pone.0287039 (PMC10310043; doi:10.1371/journal.pone.0287039)
Supplement: S2 Table — Pairwise comparisons of the species richness in the fertilization & biomass removal (+F+R), unfertilized & reduced biomass removal (-F-R), unfertilized & biomass removal (-F+R) and fertilized & reduced biomass removal (+F-R) treatment, for each region (Alb: Schwäbische Alb; Sch: Schorfheide-Chorin; Hai: Hainich-Dün), as well as for different years and seasons. Significant (< 0.05) contrasts are written in bold. (DOCX) [file pone.0287039.s013.docx]

**S2 Table: Pairwise comparison of species richness across treatments.** Pairwise comparisons of the species richness in the fertilization & biomass removal (+F+R), unfertilized & reduced biomass removal (-F-R), unfertilized & biomass removal (-F+R) and fertilized & reduced biomass removal (+F-R) treatment, for each region (Alb: Schwäbische Alb; Sch: Schorfheide-Chorin; Hai: Hainich-Dün), as well as for different years and seasons. Significant (< 0.05) contrasts are written in bold.

| **Season** | **Region** | **Contrast** | **Estimate** | **SE** | **95% CI** | **p value** |
| --- | --- | --- | --- | --- | --- | --- |
| Spring 2020 | All | +F+R vs -F-R | 0.74 | 1.00 | 1.96 | 0.88 |
|  |  | +F+R vs +F-R | 1.54 | 1.00 | 1.96 | 0.43 |
|  |  | +F+R vs -F+R | 0.48 | 1.03 | 2.02 | 0.97 |
|  |  | -F-R vs +F-R | 0.80 | 0.90 | 1.76 | 0.81 |
|  |  | -F-R vs -F+R | -0.26 | 0.94 | 1.84 | 0.99 |
|  |  | +F-R vs -F+R | -1.06 | 0.94 | 1.84 | 0.67 |
| Summer 2020 | Alb | +F+R vs -F-R | -0.17 | 1.32 | 2.59 | 1.00 |
|  |  | +F+R vs +F-R | 1.17 | 1.32 | 2.59 | 0.81 |
|  |  | +F+R vs -F+R | -1.17 | 1.32 | 2.59 | 0.81 |
|  |  | -F-R vs +F-R | 1.33 | 1.32 | 2.59 | 0.75 |
|  |  | -F-R vs -F+R | -1.00 | 1.32 | 2.59 | 0.87 |
|  |  | +F-R vs -F+R | -2.33 | 1.32 | 2.59 | 0.31 |
|  | Hai | +F+R vs -F-R | 1.67 | 1.32 | 2.59 | 0.59 |
|  |  | +F+R vs +F-R | 1.33 | 1.32 | 2.59 | 0.75 |
|  |  | +F+R vs -F+R | 0.50 | 1.32 | 2.59 | 0.98 |
|  |  | -F-R vs +F-R | -0.33 | 1.32 | 2.59 | 0.99 |
|  |  | -F-R vs -F+R | -1.17 | 1.32 | 2.59 | 0.81 |
|  |  | +F-R vs -F+R | -0.83 | 1.32 | 2.59 | 0.92 |
|  | Sch | +F+R vs -F-R | 2.75 | 1.62 | 3.18 | 0.34 |
|  |  | +F+R vs +F-R | 3.00 | 1.62 | 3.18 | 0.27 |
|  |  | +F+R vs -F+R | 1.75 | 1.62 | 3.18 | 0.70 |
|  |  | -F-R vs +F-R | 0.25 | 1.62 | 3.18 | 1.00 |
|  |  | -F-R vs -F+R | -1.00 | 1.62 | 3.18 | 0.93 |
|  |  | +F-R vs -F+R | -1.25 | 1.62 | 3.18 | 0.87 |
| Spring 2021 | Alb | +F+R vs -F-R | -0.83 | 1.45 | 2.84 | 0.94 |
|  |  | +F+R vs +F-R | -0.67 | 1.45 | 2.84 | 0.97 |
|  |  | +F+R vs -F+R | -1.83 | 1.45 | 2.84 | 0.59 |
|  |  | -F-R vs +F-R | 0.17 | 1.45 | 2.84 | 1.00 |
|  |  | -F-R vs -F+R | -1.00 | 1.45 | 2.84 | 0.90 |
|  |  | +F-R vs -F+R | -1.17 | 1.45 | 2.84 | 0.85 |
|  | Hai | +F+R vs -F-R | -0.50 | 1.45 | 2.84 | 0.99 |
|  |  | +F+R vs +F-R | 0.33 | 1.45 | 2.84 | 1.00 |
|  |  | +F+R vs -F+R | 1.83 | 1.45 | 2.84 | 0.59 |
|  |  | -F-R vs +F-R | 0.83 | 1.45 | 2.84 | 0.94 |
|  |  | -F-R vs -F+R | 2.33 | 1.45 | 2.84 | 0.38 |
|  |  | +F-R vs -F+R | 1.50 | 1.45 | 2.84 | 0.73 |
|  | Sch | +F+R vs -F-R | -0.25 | 1.77 | 3.47 | 1.00 |
|  |  | +F+R vs +F-R | 1.75 | 1.77 | 3.47 | 0.76 |
|  |  | +F+R vs -F+R | 0.25 | 1.77 | 3.47 | 1.00 |
|  |  | -F-R vs +F-R | 2.00 | 1.77 | 3.47 | 0.68 |
|  |  | -F-R vs -F+R | 0.50 | 1.77 | 3.47 | 0.99 |
|  |  | +F-R vs -F+R | -1.50 | 1.77 | 3.47 | 0.83 |
| Summer 2021 | Alb | +F+R vs -F-R | -0.67 | 1.39 | 2.72 | 0.96 |
|  |  | +F+R vs +F-R | 0.33 | 1.39 | 2.72 | 1.00 |
|  |  | +F+R vs -F+R | -2.33 | 1.39 | 2.72 | 0.35 |
|  |  | -F-R vs +F-R | 1.00 | 1.39 | 2.72 | 0.89 |
|  |  | -F-R vs -F+R | -1.67 | 1.39 | 2.72 | 0.63 |
|  |  | +F-R vs -F+R | -2.67 | 1.39 | 2.72 | 0.24 |
|  | Hai | +F+R vs -F-R | -0.67 | 1.39 | 2.72 | 0.96 |
|  |  | +F+R vs +F-R | 1.67 | 1.39 | 2.72 | 0.63 |
|  |  | +F+R vs -F+R | -1.67 | 1.39 | 2.72 | 0.63 |
|  |  | -F-R vs +F-R | 2.33 | 1.39 | 2.72 | 0.35 |
|  |  | -F-R vs -F+R | -1.00 | 1.39 | 2.72 | 0.89 |
|  |  | +F-R vs -F+R | -3.33 | 1.39 | 2.72 | 0.09 |
|  | Sch | +F+R vs -F-R | 1.50 | 1.70 | 3.33 | 0.81 |
|  |  | +F+R vs +F-R | 1.75 | 1.70 | 3.33 | 0.73 |
|  |  | +F+R vs -F+R | 1.25 | 1.70 | 3.33 | 0.88 |
|  |  | -F-R vs +F-R | 0.25 | 1.70 | 3.33 | 1.00 |
|  |  | -F-R vs -F+R | -0.25 | 1.70 | 3.33 | 1.00 |
|  |  | +F-R vs -F+R | -0.50 | 1.70 | 3.33 | 0.99 |
