# Supplementary material for: Biomass removal promotes plant diversity after short-term de-intensification of managed grasslands
Source: PLoS One. 2023 Jun 29;18(6):e0287039. doi: 10.1371/journal.pone.0287039 (PMC10310043; doi:10.1371/journal.pone.0287039)
Supplement: S3 Table — Explained variances for species richness: mar. R2 = 0.15 (adj. R2 = 0.40), standing biomass: mar. R2 = 0.23 (adj. R2 = 0.31); log(light availability): mar. R2 = 0.69 (adj. R2 = 0.69); soil moisture: mar. R2 = 0.02 (adj. R2 = 0.61). Alb: Schwäbische Alb; Sch: Schorfheide-Chorin; Hai: Hainich-Dün. (DOCX) [file pone.0287039.s014.docx]

**S3 Table: PiecewiseSEM model fit for model with main response species richness in spring** of both 2020 and 2021 with the main responses biomass removal (unfertilized & biomass removal treatment), fertilization (fertilized & reduced biomass removal treatment), standing biomass, log(light availability), background fertilization, region, sampling date and year. Explained variances for species richness: mar. R^2^ = 0.15 (adj. R^2^ = 0.40), standing biomass: mar. R^2^ = 0.23 (adj. R^2^ = 0.31); log(light availability): mar. R^2^ = 0.69 (adj. R^2^ = 0.69); soil moisture: mar. R^2^ = 0.02 (adj. R^2^ = 0.61). Alb: Schwäbische Alb; Sch: Schorfheide-Chorin; Hai: Hainich-Dün.

Fisher’s C = 15.135, df = 14, p = 0.369

| **Response** | **Predictor** | **Estimate** | **SE** | **Std. Estimate** | **p value** |
| --- | --- | --- | --- | --- | --- |
| Richness | log(Light availability) | 0.397 | 0.529 | 0.063 | 0.46 |
| Richness | Fertilization | -0.422 | 0.509 | -0.061 | 0.41 |
| Richness | Biomass removal | -0.411 | 0.555 | -0.060 | 0.46 |
| Richness | Background fertilization | -0.002 | 0.289 | -0.001 | 1.00 |
| Richness | Soil moisture | 0.164 | 0.044 | 0.366 | <0.001 |
| Standing biomass | Biomass removal | -45.805 | 8.237 | -0.463 | <0.001 |
| Standing biomass | Fertilization | 12.326 | 8.225 | 0.125 | 0.14 |
| Standing biomass | Background fertilization | 1.550 | 3.244 | 0.055 | 0.64 |
| log(Light availability) | Standing biomass | -0.008 | 0.001 | -0.746 | <0.001 |
| Soil moisture | log(Light availability) | 0.333 | 1.542 | 0.024 | 0.83 |
| Soil moisture | Standing biomass | -0.020 | 0.018 | -0.126 | 0.27 |
