# Supplementary material for: Biomass removal promotes plant diversity after short-term de-intensification of managed grasslands
Source: PLoS One. 2023 Jun 29;18(6):e0287039. doi: 10.1371/journal.pone.0287039 (PMC10310043; doi:10.1371/journal.pone.0287039)
Supplement: S4 Table — Explained variances for Shannon diversity: mar. R2 = 0.14 (adj. R2 = 0.35), standing biomass: mar. R2 = 0.23 (adj. R2 = 0.31); log(light availability): mar. R2 = 0.69 (adj. R2 = 0.69); soil moisture: mar. R2 = 0.02 (adj. R2 = 0.61). Alb: Schwäbische Alb; Sch: Schorfheide-Chorin; Hai: Hainich-Dün. (DOCX) [file pone.0287039.s015.docx]

**S4 Table: PiecewiseSEM model fit for model with main response Shannon diversity in spring** of both 2020 and 2021 with the main responses biomass removal (unfertilized *&* biomass removal treatment), fertilization (fertilized *&* reduced biomass removal treatment), standing biomass, log(light availability), background fertilization region, sampling date and year. Explained variances for Shannon diversity: mar. R^2^ = 0.14 (adj. R^2^ = 0.39), standing biomass: mar. R^2^ = 0.23 (adj. R^2^ = 0.31); log(light availability): mar. R^2^ = 0.69 (adj. R^2^ = 0.69); soil moisture: mar. R^2^ = 0.02 (adj. R^2^ = 0.61). Alb: Schwäbische Alb; Sch: Schorfheide-Chorin; Hai: Hainich-Dün.

Fisher’s C = 17.263, df = 14, p = 0.242

| **Response** | **Predictor** | **Estimate** | **SE** | **Std. Estimate** | **p value** |
| --- | --- | --- | --- | --- | --- |
| Diversity | log(Light availability) | 0.394 | 0.400 | 0.085 | 0.33 |
| Diversity | Fertilization | -0.381 | 0.384 | -0.075 | 0.32 |
| Diversity | Biomass removal | -0.415 | 0.419 | -0.082 | 0.32 |
| Diversity | Background fertilization | 0.000 | 0.218 | 0.000 | 1.00 |
| Diversity | Soil moisture | 0.116 | 0.033 | 0.349 | <0.001 |
| Standing biomass | Biomass removal | -45.805 | 8.237 | -0.463 | <0.001 |
| Standing biomass | Fertilization | 12.326 | 8.225 | 0.125 | 0.14 |
| Standing biomass | Background fertilization | 1.550 | 3.244 | 0.055 | 0.64 |
| log(Light availability) | Standing biomass | -0.008 | 0.001 | -0.746 | <0.001 |
| Soil moisture | log(Light availability) | 0.333 | 1.542 | 0.024 | 0.83 |
| Soil moisture | Standing biomass | -0.020 | 0.018 | -0.126 | 0.27 |
