# Supplementary material for: Biomass removal promotes plant diversity after short-term de-intensification of managed grasslands
Source: PLoS One. 2023 Jun 29;18(6):e0287039. doi: 10.1371/journal.pone.0287039 (PMC10310043; doi:10.1371/journal.pone.0287039)
Supplement: S5 Table — Explained variances for species richness: mar. R2 = 0.11 (adj. R2 = 0.56), standing biomass: mar. R2 = 0.35 (adj. R2 = 0.69); biomass production: mar. R2 = 0.32 (adj. R2 = 0.43); log(light availability): mar. R2 = 0.66 (adj. R2 = 0.67); soil moisture: mar. R2 = 0.02 (adj. R2 = 0.74). Alb: Schwäbische Alb; Sch: Schorfheide-Chorin; Hai: Hainich-Dün. (DOCX) [file pone.0287039.s016.docx]

**S5 Table: PiecewiseSEM model fit for model with main response species richness in summer** of both 2020 and 2021 with the main responses biomass removal (unfertilized *&* biomass removal treatment), fertilization (fertilized *&* reduced biomass removal treatment), standing biomass, biomass production, log(light availability), background fertilization region, sampling date and year. Explained variances for species richness: mar. R^2^ = 0.11 (adj. R^2^ = 0.56), standing biomass: mar. R^2^ = 0.35 (adj. R^2^ = 0.69); biomass production: mar. R^2^ = 0.32 (adj. R^2^ = 0.43); log(light availability): mar. R^2^ = 0.66 (adj. R^2^ = 0.67); soil moisture: mar. R^2^ = 0.02 (adj. R^2^ = 0.74). Alb: Schwäbische Alb; Sch: Schorfheide-Chorin; Hai: Hainich-Dün.

Fisher’s C = 24.063, df = 22, p = 0.344

| **Response** | **Predictor** | **Estimate** | **SE** | **Std. Estimate** | **p value** |
| --- | --- | --- | --- | --- | --- |
| Richness | log(Light availability) | -0.471 | 0.318 | -0.135 | 0.14 |
| Richness | Fertilization | -0.913 | 0.461 | -0.125 | 0.05 |
| Richness | Biomass removal | 1.501 | 0.596 | 0.205 | 0.01 |
| Richness | Background fertilization | -0.165 | 0.404 | -0.076 | 0.69 |
| Richness | Soil moisture | 0.109 | 0.038 | 0.299 | 0.01 |
| Standing biomass | Biomass removal | -142.119 | 13.516 | -0.735 | <0.001 |
| Standing biomass | Biomass production | 0.131 | 0.042 | 0.229 | <0.001 |
| Biomass production | Fertilization | 18.966 | 24.192 | 0.056 | 0.43 |
| Biomass production | Biomass removal | 192.231 | 24.214 | 0.567 | <0.001 |
| Biomass production | Background fertilization | 7.209 | 11.003 | 0.072 | 0.52 |
| log(Light availability) | Standing biomass | -0.009 | 0.001 | -0.815 | <0.001 |
| Soil moisture | log(Light availability) | -0.946 | 0.929 | -0.099 | 0.31 |
| Soil moisture | Standing biomass | -0.025 | 0.011 | -0.242 | 0.02 |
| ~~Soil moisture | ~~Biomass production | 0.022 | - | 0.022 | 0.41 |
