# Supplementary material for: Biomass removal promotes plant diversity after short-term de-intensification of managed grasslands
Source: PLoS One. 2023 Jun 29;18(6):e0287039. doi: 10.1371/journal.pone.0287039 (PMC10310043; doi:10.1371/journal.pone.0287039)
Supplement: S8 Table — (DOCX) [file pone.0287039.s019.docx]

**S8 Table: Permutation test of fitted vectors of the environmental variables (standing biomass, light availability and soil moisture), plant species richness and Shannon diversity in 2021** on the NMDS ordination (NMDS1 and NMDS2) for spring and summer of 2021 across all regions (Schwäbische Alb, Hainich-Dün, Schorfheide-Chorin) (5 Fig).

| **Season** | **Factor** | **NMDS1** | **NMDS2** | **R2** | **p value** |
| --- | --- | --- | --- | --- | --- |
| Spring | Standing biomass | -0.83 | 0.56 | 0.05 | 0.21 |
|  | Soil moisture | 0.91 | 0.42 | 0.01 | 0.77 |
|  | Light availability | 0.99 | -0.17 | 0.07 | 0.10 |
|  | Richness | -0.01 | 1.00 | 0.06 | 0.16 |
|  | Diversity | 0.27 | 0.96 | 0.06 | 0.17 |
| Summer | Standing biomass | 0.10 | -0.99 | 0.02 | 0.48 |
|  | Soil moisture | 0.78 | 0.63 | 0.10 | 0.05 |
|  | Light availability | -0.58 | 0.81 | 0.02 | 0.48 |
|  | Richness | 0.56 | 0.83 | 0.13 | 0.01 |
|  | Diversity | 0.59 | 0.80 | 0.20 | <0.001 |
