# Supplementary material for: Biomass removal promotes plant diversity after short-term de-intensification of managed grasslands
Source: PLoS One. 2023 Jun 29;18(6):e0287039. doi: 10.1371/journal.pone.0287039 (PMC10310043; doi:10.1371/journal.pone.0287039)
Supplement: S9 Table — Linear mixed effect model showing the effect of the unfertilized & reduced biomass removal (-F-R), fertilized & reduced biomass removal (+F-R), unfertilized & biomass removal (-F+R) on Shannon diversity in comparison with the fertilized & biomass removal (+F+R) treatment for each regions (Alb: Schwäbische Alb; Sch: Schorfheide-Chorin; Hai: Hainich-Dün), as well as for different years and seasons. (DOCX) [file pone.0287039.s020.docx]

**S9 Table: Shannon diversity in response to treatments.** Linear mixed effect model showing the effect of the unfertilized & reduced biomass removal (-F-R), fertilized & reduced biomass removal (+F-R), unfertilized & biomass removal (-F+R) on Shannon diversity in comparison with the fertilized & biomass removal (+F+R) treatment for each regions (Alb: Schwäbische Alb; Sch: Schorfheide-Chorin; Hai: Hainich-Dün), as well as for different years and seasons. Note that rows are relative to the intercept.

| **Season** | **Predictor** | **Estimate** | **SE** | **95% CI** | **p value** |
| --- | --- | --- | --- | --- | --- |
| Spring 2020 | Intercept (Alb) | 11.73 | 0.83 | 1.63 | <0.001 |
|  | Hai | -7.23 | 0.98 | 1.92 | <0.001 |
|  | Sch | -5.30 | 1.10 | 2.16 | <0.001 |
|  | -F-R | -0.26 | 0.79 | 1.55 | 0.74 |
|  | +F-R | -1.05 | 0.79 | 1.55 | 0.19 |
|  | -F+R | -0.64 | 0.81 | 1.59 | 0.44 |
| Summer 2020 | Intercept (Alb) | 7.55 | 0.97 | 1.90 | <0.001 |
|  | Hai | -1.18 | 1.37 | 2.69 | 0.40 |
|  | Sch | -0.08 | 1.53 | 3.00 | 0.96 |
|  | -F-R | -1.40 | 1.00 | 1.96 | 0.17 |
|  | +F-R | -0.88 | 1.00 | 1.96 | 0.39 |
|  | -F+R | 3.23 | 1.00 | 1.96 | <0.001 |
|  | -F-R : Hai | 0.61 | 1.42 | 2.78 | 0.67 |
|  | +F-R : Hai | 0.03 | 1.42 | 2.78 | 0.98 |
|  | -F+R : Hai | -3.89 | 1.42 | 2.78 | 0.01 |
|  | -F-R : Sch | 0.94 | 1.59 | 3.12 | 0.56 |
|  | +F-R : Sch | -0.40 | 1.59 | 3.12 | 0.80 |
|  | -F+R : Sch | -3.74 | 1.59 | 3.12 | 0.02 |
| Spring 2021 | Intercept (Alb) | 9.68 | 1.07 | 2.10 | <0.001 |
|  | Hai | -3.77 | 1.51 | 2.96 | 0.02 |
|  | Sch | -0.72 | 1.69 | 3.31 | 0.67 |
|  | -F-R | 0.03 | 0.99 | 1.94 | 0.98 |
|  | +F-R | -0.13 | 0.99 | 1.94 | 0.90 |
|  | -F+R | 1.43 | 0.99 | 1.94 | 0.16 |
|  | -F-R : Hai | 0.58 | 1.40 | 2.74 | 0.68 |
|  | +F-R : Hai | 1.37 | 1.40 | 2.74 | 0.34 |
|  | -F+R : Hai | -2.34 | 1.40 | 2.74 | 0.10 |
|  | -F-R : Sch | 1.09 | 1.57 | 3.08 | 0.49 |
|  | +F-R : Sch | -1.35 | 1.57 | 3.08 | 0.39 |
|  | -F+R : Sch | -1.03 | 1.57 | 3.08 | 0.52 |
| Summer 2021 | Intercept (Alb) | 9.01 | 1.10 | 2.16 | <0.001 |
|  | Hai | -1.89 | 1.56 | 3.06 | 0.24 |
|  | Sch | -1.35 | 1.75 | 3.43 | 0.45 |
|  | -F-R | -0.04 | 1.01 | 1.98 | 0.97 |
|  | +F-R | -1.11 | 1.01 | 1.98 | 0.28 |
|  | -F+R | 0.24 | 1.01 | 1.98 | 0.81 |
|  | -F-R : Hai | 1.15 | 1.43 | 2.80 | 0.43 |
|  | +F-R : Hai | 1.27 | 1.43 | 2.80 | 0.38 |
|  | -F+R : Hai | 0.93 | 1.43 | 2.80 | 0.52 |
|  | -F-R : Sch | -0.08 | 1.60 | 3.14 | 0.96 |
|  | +F-R : Sch | 0.65 | 1.60 | 3.14 | 0.69 |
|  | -F+R : Sch | -0.58 | 1.60 | 3.14 | 0.72 |
