# Supplementary material for: Biomass removal promotes plant diversity after short-term de-intensification of managed grasslands
Source: PLoS One. 2023 Jun 29;18(6):e0287039. doi: 10.1371/journal.pone.0287039 (PMC10310043; doi:10.1371/journal.pone.0287039)
Supplement: S10 Table — Pairwise comparisons of the Shannon diversity in the fertilization & biomass removal (+F+R), unfertilized & reduced biomass removal (-F-R), unfertilized & biomass removal (-F+R) and fertilized & reduced biomass removal (+F-R) treatment, for each region (Alb: Schwäbische Alb; Sch: Schorfheide-Chorin; Hai: Hainich-Dün), as well as for different years and seasons. No pairwise comparison shown for spring 2021, as the predictor ‘treatment’ was not part of the most parsimonious model. Significant (< 0.05) contrasts are written in bold. (DOCX) [file pone.0287039.s021.docx]

**S10 Table: Pairwise comparison of Shannon diversity across treatments.** Pairwise comparisons of the Shannon diversity in the fertilization & biomass removal (+F+R), unfertilized & reduced biomass removal (-F-R), unfertilized & biomass removal (-F+R) and fertilized & reduced biomass removal (+F-R) treatment, for each region (Alb: Schwäbische Alb; Sch: Schorfheide-Chorin; Hai: Hainich-Dün), as well as for different years and seasons. Significant (< 0.05) contrasts are written in bold.

| **Season** | **Region** | **Contrast** | **Estimate** | **SE** | **95% CI** | **p value** |
| --- | --- | --- | --- | --- | --- | --- |
| Spring 2020 | All | +F+R vs -F-R | 0.26 | 0.79 | 1.55 | 0.99 |
|  |  | +F+R vs +F-R | 1.05 | 0.79 | 1.55 | 0.55 |
|  |  | +F+R vs -F+R | 0.64 | 0.81 | 1.59 | 0.86 |
|  |  | -F-R vs +F-R | 0.79 | 0.71 | 1.39 | 0.68 |
|  |  | -F-R vs -F+R | 0.37 | 0.74 | 1.45 | 0.96 |
|  |  | +F-R vs -F+R | -0.41 | 0.74 | 1.45 | 0.94 |
| Summer 2020 | Alb | +F+R vs -F-R | 1.40 | 1.00 | 1.96 | 0.51 |
|  |  | +F+R vs +F-R | 0.88 | 1.00 | 1.96 | 0.82 |
|  |  | +F+R vs -F+R | -3.23 | 1.00 | 1.96 | 0.01 |
|  |  | -F-R vs +F-R | -0.52 | 1.00 | 1.96 | 0.95 |
|  |  | -F-R vs -F+R | -4.64 | 1.00 | 1.96 | **<0.001** |
|  |  | +F-R vs -F+R | -4.12 | 1.00 | 1.96 | **<0.001** |
|  | Hai | +F+R vs -F-R | 0.80 | 1.00 | 1.96 | 0.86 |
|  |  | +F+R vs +F-R | 0.85 | 1.00 | 1.96 | 0.83 |
|  |  | +F+R vs -F+R | 0.66 | 1.00 | 1.96 | 0.91 |
|  |  | -F-R vs +F-R | 0.05 | 1.00 | 1.96 | 1.00 |
|  |  | -F-R vs -F+R | -0.14 | 1.00 | 1.96 | 1.00 |
|  |  | +F-R vs -F+R | -0.19 | 1.00 | 1.96 | 1.00 |
|  | Sch | +F+R vs -F-R | 0.47 | 1.23 | 2.41 | 0.98 |
|  |  | +F+R vs +F-R | 1.28 | 1.23 | 2.41 | 0.73 |
|  |  | +F+R vs -F+R | 0.51 | 1.23 | 2.41 | 0.98 |
|  |  | -F-R vs +F-R | 0.82 | 1.23 | 2.41 | 0.91 |
|  |  | -F-R vs -F+R | 0.04 | 1.23 | 2.41 | 1.00 |
|  |  | +F-R vs -F+R | -0.77 | 1.23 | 2.41 | 0.92 |
| Spring 2021 | Alb | +F+R vs -F-R | -0.03 | 0.99 | 1.94 | 1.00 |
|  |  | +F+R vs +F-R | 0.13 | 0.99 | 1.94 | 1.00 |
|  |  | +F+R vs -F+R | -1.43 | 0.99 | 1.94 | 0.48 |
|  |  | -F-R vs +F-R | 0.16 | 0.99 | 1.94 | 1.00 |
|  |  | -F-R vs -F+R | -1.40 | 0.99 | 1.94 | 0.50 |
|  |  | +F-R vs -F+R | -1.56 | 0.99 | 1.94 | 0.41 |
|  | Hai | +F+R vs -F-R | -0.61 | 0.99 | 1.94 | 0.93 |
|  |  | +F+R vs +F-R | -1.24 | 0.99 | 1.94 | 0.60 |
|  |  | +F+R vs -F+R | 0.92 | 0.99 | 1.94 | 0.79 |
|  |  | -F-R vs +F-R | -0.63 | 0.99 | 1.94 | 0.92 |
|  |  | -F-R vs -F+R | 1.52 | 0.99 | 1.94 | 0.43 |
|  |  | +F-R vs -F+R | 2.15 | 0.99 | 1.94 | 0.15 |
|  | Sch | +F+R vs -F-R | -1.12 | 1.21 | 2.37 | 0.79 |
|  |  | +F+R vs +F-R | 1.48 | 1.21 | 2.37 | 0.62 |
|  |  | +F+R vs -F+R | -0.40 | 1.21 | 2.37 | 0.99 |
|  |  | -F-R vs +F-R | 2.60 | 1.21 | 2.37 | 0.16 |
|  |  | -F-R vs -F+R | 0.72 | 1.21 | 2.37 | 0.93 |
|  |  | +F-R vs -F+R | -1.88 | 1.21 | 2.37 | 0.42 |
| Summer 2021 | Alb | +F+R vs -F-R | 0.04 | 1.01 | 1.98 | 1.00 |
|  |  | +F+R vs +F-R | 1.11 | 1.01 | 1.98 | 0.69 |
|  |  | +F+R vs -F+R | -0.24 | 1.01 | 1.98 | 1.00 |
|  |  | -F-R vs +F-R | 1.07 | 1.01 | 1.98 | 0.72 |
|  |  | -F-R vs -F+R | -0.28 | 1.01 | 1.98 | 0.99 |
|  |  | +F-R vs -F+R | -1.35 | 1.01 | 1.98 | 0.55 |
|  | Hai | +F+R vs -F-R | -1.10 | 1.01 | 1.98 | 0.70 |
|  |  | +F+R vs +F-R | -0.16 | 1.01 | 1.98 | 1.00 |
|  |  | +F+R vs -F+R | -1.17 | 1.01 | 1.98 | 0.66 |
|  |  | -F-R vs +F-R | 0.95 | 1.01 | 1.98 | 0.79 |
|  |  | -F-R vs -F+R | -0.06 | 1.01 | 1.98 | 1.00 |
|  |  | +F-R vs -F+R | -1.01 | 1.01 | 1.98 | 0.75 |
|  | Sch | +F+R vs -F-R | 0.12 | 1.24 | 2.43 | 1.00 |
|  |  | +F+R vs +F-R | 0.46 | 1.24 | 2.43 | 0.98 |
|  |  | +F+R vs -F+R | 0.34 | 1.24 | 2.43 | 0.99 |
|  |  | -F-R vs +F-R | 0.33 | 1.24 | 2.43 | 0.99 |
|  |  | -F-R vs -F+R | 0.22 | 1.24 | 2.43 | 1.00 |
|  |  | +F-R vs -F+R | -0.12 | 1.24 | 2.43 | 1.00 |
