# Supplementary material for: Biomass removal promotes plant diversity after short-term de-intensification of managed grasslands
Source: PLoS One. 2023 Jun 29;18(6):e0287039. doi: 10.1371/journal.pone.0287039 (PMC10310043; doi:10.1371/journal.pone.0287039)
Supplement: S11 Table — Linear mixed effect model showing the effect of the unfertilized & reduced biomass removal (-F-R), fertilized & reduced biomass removal (+F-R), unfertilized & biomass removal (-F+R) on standing biomass in comparison with the fertilized & biomass removal treatment for each regions (Alb: Schwäbische Alb; Sch: Schorfheide-Chorin; Hai: Hainich-Dün), as well as for different years and seasons. (DOCX) [file pone.0287039.s022.docx]

**S11 Table: Standing biomass in response to treatments.** Linear mixed effect model showing the effect of the unfertilized & reduced biomass removal (-F-R), fertilized & reduced biomass removal (+F-R), unfertilized & biomass removal (-F+R) on standing biomass in comparison with the fertilized & biomass removal treatment for each regions (Alb: Schwäbische Alb; Sch: Schorfheide-Chorin; Hai: Hainich-Dün), as well as for different years and seasons. Note that rows are relative to the intercept.

| **Season** | **Predictor** | **Estimate** | **SE** | **95% CI** | **p value** |
| --- | --- | --- | --- | --- | --- |
| Spring 2020 | Intercept (Alb) | 195.81 | 22.10 | 43.32 | < 0.001 |
|  | Hai | -157.18 | 32.78 | 64.25 | < 0.001 |
|  | Sch | -100.39 | 38.22 | 74.91 | 0.01 |
|  | -F-R | -5.43 | 29.03 | 56.90 | 0.85 |
|  | +F-R | 20.99 | 29.03 | 56.90 | 0.48 |
|  | -F+R | -34.01 | 29.03 | 56.90 | 0.25 |
|  | -F-R : Hai | 7.60 | 43.06 | 84.40 | 0.86 |
|  | +F-R : Hai | -3.62 | 43.06 | 84.40 | 0.93 |
|  | -F+R : Hai | 35.27 | 44.65 | 87.51 | 0.44 |
|  | -F-R : Sch | 67.66 | 50.28 | 98.55 | 0.19 |
|  | +F-R : Sch | 15.91 | 48.44 | 94.94 | 0.74 |
| Summer 2020 | Intercept (Alb) | 65.19 | 14.28 | 27.99 | < 0.001 |
|  | Hai | 1.81 | 20.19 | 39.57 | 0.93 |
|  | Sch | -21.35 | 22.57 | 44.24 | 0.35 |
|  | -F-R | 125.92 | 17.36 | 34.03 | < 0.001 |
|  | +F-R | 150.16 | 17.36 | 34.03 | < 0.001 |
|  | -F+R | -22.80 | 17.36 | 34.03 | 0.20 |
|  | -F-R : Hai | -45.59 | 24.55 | 48.12 | 0.07 |
|  | +F-R : Hai | -56.08 | 24.55 | 48.12 | 0.03 |
|  | -F+R : Hai | 32.93 | 24.55 | 48.12 | 0.19 |
|  | -F-R : Sch | 121.58 | 27.45 | 53.80 | < 0.001 |
|  | +F-R : Sch | 98.96 | 27.45 | 53.80 | < 0.001 |
|  | -F+R : Sch | 30.39 | 27.45 | 53.80 | 0.27 |
| Spring 2021 | Intercept (Alb) | 53.25 | 12.19 | 23.89 | < 0.001 |
|  | Hai | -3.62 | 17.24 | 33.79 | 0.83 |
|  | Sch | 18.27 | 19.27 | 37.77 | 0.35 |
|  | -F-R | 9.77 | 13.74 | 26.93 | 0.48 |
|  | +F-R | 26.41 | 13.74 | 26.93 | 0.06 |
|  | -F+R | 0.00 | 13.74 | 26.93 | 1.00 |
|  | -F-R : Hai | -17.01 | 19.43 | 38.08 | 0.39 |
|  | +F-R : Hai | 3.62 | 19.43 | 38.08 | 0.85 |
|  | -F+R : Hai | -11.94 | 19.43 | 38.08 | 0.54 |
|  | -F-R : Sch | 133.00 | 21.72 | 42.57 | < 0.001 |
|  | +F-R : Sch | 78.34 | 21.72 | 42.57 | < 0.001 |
|  | -F+R : Sch | -26.05 | 21.72 | 42.57 | 0.24 |
| Summer 2021 | Intercept (Alb) | 140.45 | 33.09 | 64.86 | < 0.001 |
|  | Hai | 71.28 | 46.80 | 91.73 | 0.14 |
|  | Sch | 99.87 | 52.33 | 102.57 | 0.06 |
|  | -F-R | 108.19 | 36.24 | 71.03 | < 0.001 |
|  | +F-R | 93.35 | 36.24 | 71.03 | 0.01 |
|  | -F+R | -5.07 | 36.24 | 71.03 | 0.89 |
|  | -F-R : Hai | -78.16 | 51.25 | 100.45 | 0.14 |
|  | +F-R : Hai | -34.74 | 51.25 | 100.45 | 0.50 |
|  | -F+R : Hai | -39.80 | 51.25 | 100.45 | 0.44 |
|  | -F-R : Sch | -3.44 | 57.30 | 112.31 | 0.95 |
|  | +F-R : Sch | 73.27 | 57.30 | 112.31 | 0.21 |
|  | -F+R : Sch | -11.22 | 57.30 | 112.31 | 0.85 |
