# Supplementary material for: Biomass removal promotes plant diversity after short-term de-intensification of managed grasslands
Source: PLoS One. 2023 Jun 29;18(6):e0287039. doi: 10.1371/journal.pone.0287039 (PMC10310043; doi:10.1371/journal.pone.0287039)
Supplement: S12 Table — Pairwise comparisons of the standing biomass in the fertilization & biomass removal, unfertilized & reduced biomass removal, unfertilized & biomass removal and fertilized & reduced biomass removal treatment, for each region (Alb: Schwäbische Alb; Sch: Schorfheide-Chorin; Hai: Hainich-Dün), as well as for different years and seasons. Significant (< 0.05) contrasts are written in bold. Due to missing data on fertilized & biomass removal treatments in spring 2020 for the Schorfheide-Chorin no pairwise contrasts shown (*). (DOCX) [file pone.0287039.s023.docx]

**S12 Table: Pairwise comparison of standing biomass across treatments** Pairwise comparisons of the standing biomass in the fertilization & biomass removal, unfertilized & reduced biomass removal, unfertilized & biomass removal and fertilized & reduced biomass removal treatment, for each region (Alb: Schwäbische Alb; Sch: Schorfheide-Chorin; Hai: Hainich-Dün), as well as for different years and seasons. Significant (< 0.05) contrasts are written in bold. Due to missing data on fertilized & biomass removal treatments in spring 2020 for the Schorfheide-Chorin no pairwise contrasts shown (*).

| **Season** | **Region** | **Contrast** | **Estimate** | **SE** | **95% CI** | **p value** |
| --- | --- | --- | --- | --- | --- | --- |
| Spring 2020 | Alb | +F+R vs -F-R | 5.43 | 29.00 | 56.84 | 1.00 |
|  |  | +F+R vs +F-R | -20.99 | 29.00 | 56.84 | 0.89 |
|  |  | +F+R vs -F+R | 34.01 | 29.00 | 56.84 | 0.65 |
|  |  | -F-R vs +F-R | -26.41 | 29.00 | 56.84 | 0.80 |
|  |  | -F-R vs -F+R | 28.59 | 29.00 | 56.84 | 0.76 |
|  |  | +F-R vs -F+R | 55.00 | 29.00 | 56.84 | 0.25 |
|  | Hai | +F+R vs -F-R | -2.17 | 31.80 | 62.33 | 1.00 |
|  |  | +F+R vs +F-R | -17.37 | 31.80 | 62.33 | 0.95 |
|  |  | +F+R vs -F+R | -1.26 | 34.10 | 66.84 | 1.00 |
|  |  | -F-R vs +F-R | -15.20 | 31.80 | 62.33 | 0.96 |
|  |  | -F-R vs -F+R | 0.91 | 34.10 | 66.84 | 1.00 |
|  |  | +F-R vs -F+R | 16.11 | 34.10 | 66.84 | 0.96 |
|  | Sch | +F+R vs -F-R | nonEst* | NA | NA | NA |
|  |  | +F+R vs +F-R | nonEst* | NA | NA | NA |
|  |  | +F+R vs -F+R | nonEst* | NA | NA | NA |
|  |  | -F-R vs +F-R | 25.34 | 38.90 | 76.24 | 0.91 |
|  |  | -F-R vs -F+R | 96.25 | 41.10 | 80.56 | 0.11 |
|  |  | +F-R vs -F+R | 70.90 | 38.90 | 76.24 | 0.28 |
| Summer 2020 | Alb | +F+R vs -F-R | -125.92 | 17.40 | 34.10 | **< 0.001** |
|  |  | +F+R vs +F-R | -150.16 | 17.40 | 34.10 | **< 0.001** |
|  |  | +F+R vs -F+R | 22.80 | 17.40 | 34.10 | 0.56 |
|  |  | -F-R vs +F-R | -24.24 | 17.40 | 34.10 | 0.51 |
|  |  | -F-R vs -F+R | 148.71 | 17.40 | 34.10 | **< 0.001** |
|  |  | +F-R vs -F+R | 172.96 | 17.40 | 34.10 | **< 0.001** |
|  | Hai | +F+R vs -F-R | -80.33 | 17.40 | 34.10 | **< 0.001** |
|  |  | +F+R vs +F-R | -94.08 | 17.40 | 34.10 | **< 0.001** |
|  |  | +F+R vs -F+R | -10.13 | 17.40 | 34.10 | 0.94 |
|  |  | -F-R vs +F-R | -13.75 | 17.40 | 34.10 | 0.86 |
|  |  | -F-R vs -F+R | 70.20 | 17.40 | 34.10 | **< 0.001** |
|  |  | +F-R vs -F+R | 83.95 | 17.40 | 34.10 | **< 0.001** |
|  | Sch | +F+R vs -F-R | -247.49 | 21.30 | 41.75 | **< 0.001** |
|  |  | +F+R vs +F-R | -249.12 | 21.30 | 41.75 | **< 0.001** |
|  |  | +F+R vs -F+R | -7.60 | 21.30 | 41.75 | 0.98 |
|  |  | -F-R vs +F-R | -1.63 | 21.30 | 41.75 | 1.00 |
|  |  | -F-R vs -F+R | 239.90 | 21.30 | 41.75 | **< 0.001** |
|  |  | +F-R vs -F+R | 241.52 | 21.30 | 41.75 | **< 0.001** |
| Spring 2021 | Alb | +F+R vs -F-R | -9.77 | 13.70 | 26.85 | 0.89 |
|  |  | +F+R vs +F-R | -26.41 | 13.70 | 26.85 | 0.24 |
|  |  | +F+R vs -F+R | 0.00 | 13.70 | 26.85 | 1.00 |
|  |  | -F-R vs +F-R | -16.64 | 13.70 | 26.85 | 0.62 |
|  |  | -F-R vs -F+R | 9.77 | 13.70 | 26.85 | 0.89 |
|  |  | +F-R vs -F+R | 26.41 | 13.70 | 26.85 | 0.24 |
|  | Hai | +F+R vs -F-R | 7.24 | 13.70 | 26.85 | 0.95 |
|  |  | +F+R vs +F-R | -30.03 | 13.70 | 26.85 | 0.15 |
|  |  | +F+R vs -F+R | 11.94 | 13.70 | 26.85 | 0.82 |
|  |  | -F-R vs +F-R | -37.27 | 13.70 | 26.85 | 0.05 |
|  |  | -F-R vs -F+R | 4.70 | 13.70 | 26.85 | 0.99 |
|  |  | +F-R vs -F+R | 41.97 | 13.70 | 26.85 | 0.02 |
|  | Sch | +F+R vs -F-R | -142.74 | 16.80 | 32.93 | **< 0.001** |
|  |  | +F+R vs +F-R | -104.75 | 16.80 | 32.93 | **< 0.001** |
|  |  | +F+R vs -F+R | 26.05 | 16.80 | 32.93 | 0.42 |
|  |  | -F-R vs +F-R | 37.99 | 16.80 | 32.93 | 0.13 |
|  |  | -F-R vs -F+R | 168.79 | 16.80 | 32.93 | **< 0.001** |
|  |  | +F-R vs -F+R | 130.80 | 16.80 | 32.93 | **< 0.001** |
| Summer 2021 | Alb | +F+R vs -F-R | -108.19 | 36.20 | 70.95 | **0.02** |
|  |  | +F+R vs +F-R | -93.35 | 36.20 | 70.95 | 0.06 |
|  |  | +F+R vs -F+R | 5.07 | 36.20 | 70.95 | 1.00 |
|  |  | -F-R vs +F-R | 14.84 | 36.20 | 70.95 | 0.98 |
|  |  | -F-R vs -F+R | 113.25 | 36.20 | 70.95 | **0.02** |
|  |  | +F-R vs -F+R | 98.42 | 36.20 | 70.95 | **0.05** |
|  | Hai | +F+R vs -F-R | -30.03 | 36.20 | 70.95 | 0.84 |
|  |  | +F+R vs +F-R | -58.62 | 36.20 | 70.95 | 0.38 |
|  |  | +F+R vs -F+R | 44.87 | 36.20 | 70.95 | 0.61 |
|  |  | -F-R vs +F-R | -28.58 | 36.20 | 70.95 | 0.86 |
|  |  | -F-R vs -F+R | 74.90 | 36.20 | 70.95 | 0.18 |
|  |  | +F-R vs -F+R | 103.48 | 36.20 | 70.95 | **0.03** |
|  | Sch | +F+R vs -F-R | -104.75 | 44.40 | 87.02 | 0.10 |
|  |  | +F+R vs +F-R | -166.62 | 44.40 | 87.02 | **< 0.001** |
|  |  | +F+R vs -F+R | 16.28 | 44.40 | 87.02 | 0.98 |
|  |  | -F-R vs +F-R | -61.87 | 44.40 | 87.02 | 0.51 |
|  |  | -F-R vs -F+R | 121.03 | 44.40 | 87.02 | **0.05** |
|  |  | +F-R vs -F+R | 182.91 | 44.40 | 87.02 | **< 0.001** |
