# Supplementary material for: Biomass removal promotes plant diversity after short-term de-intensification of managed grasslands
Source: PLoS One. 2023 Jun 29;18(6):e0287039. doi: 10.1371/journal.pone.0287039 (PMC10310043; doi:10.1371/journal.pone.0287039)
Supplement: S13 Table — Linear mixed effect model showing the effect of the unfertilized & reduced biomass removal (-F-R), fertilized & reduced biomass removal (+F-R), unfertilized & biomass removal (-F+R) on biomass production in comparison with the fertilized & biomass removal treatment for each regions (Alb: Schwäbische Alb; Sch: Schorfheide-Chorin; Hai: Hainich-Dün), in summer for all different years. (DOCX) [file pone.0287039.s024.docx]

**S13 Table: Biomass production in response to treatments.** Linear mixed effect model showing the effect of the unfertilized & reduced biomass removal (-F-R), fertilized & reduced biomass removal (+F-R), unfertilized & biomass removal (-F+R) on biomass production in comparison with the fertilized & biomass removal treatment for each regions (Alb: Schwäbische Alb; Sch: Schorfheide-Chorin; Hai: Hainich-Dün), in summer for all different years. Note that rows are relative to the intercept.

| **Season** | **Predictor** | **Estimate** | **SE** | **95% CI** | **p value** |
| --- | --- | --- | --- | --- | --- |
| Summer 2020 | Intercept (Alb) | 227.34 | 57.14 | 111.99 | < 0.001 |
|  | Hai | 95.19 | 84.75 | 166.11 | 0.27 |
|  | Sch | -282.08 | 98.61 | 193.28 | 0.01 |
|  | -F-R | -226.61 | 71.98 | 141.08 | < 0.001 |
|  | +F-R | -228.78 | 71.98 | 141.08 | < 0.001 |
|  | -F+R | 11.22 | 71.98 | 141.08 | 0.88 |
|  | -F-R : Hai | 1.78 | 106.76 | 209.25 | 0.99 |
|  | +F-R : Hai | 11.77 | 106.76 | 209.25 | 0.91 |
|  | -F+R : Hai | -5.19 | 110.86 | 217.29 | 0.96 |
|  | -F-R : Sch | 429.38 | 124.67 | 244.35 | < 0.001 |
|  | +F-R : Sch | 444.18 | 120.48 | 236.14 | < 0.001 |
| Summer 2021 | Intercept (Alb) | 481.09 | 55.61 | 109.00 | < 0.001 |
|  | Hai | -96.65 | 78.65 | 154.15 | 0.23 |
|  | Sch | -163.15 | 87.93 | 172.34 | 0.07 |
|  | -F-R | -295.47 | 64.70 | 126.81 | < 0.001 |
|  | +F-R | -326.95 | 64.70 | 126.81 | < 0.001 |
|  | -F+R | -5.07 | 64.70 | 126.81 | 0.94 |
|  | -F-R : Hai | 110.40 | 91.49 | 179.32 | 0.23 |
|  | +F-R : Hai | 133.19 | 91.49 | 179.32 | 0.15 |
|  | -F+R : Hai | -27.86 | 91.49 | 179.32 | 0.76 |
|  | -F-R : Sch | 108.33 | 102.29 | 200.49 | 0.30 |
|  | +F-R : Sch | 239.67 | 102.29 | 200.49 | 0.02 |
|  | -F+R : Sch | 14.84 | 102.29 | 200.49 | 0.89 |
