# Supplementary material for: Biomass removal promotes plant diversity after short-term de-intensification of managed grasslands
Source: PLoS One. 2023 Jun 29;18(6):e0287039. doi: 10.1371/journal.pone.0287039 (PMC10310043; doi:10.1371/journal.pone.0287039)
Supplement: S14 Table — Pairwise comparisons of biomass production in the fertilization & biomass removal (+F+R), unfertilized & reduced biomass removal (-F-R), unfertilized & biomass removal (-F+R) and fertilized & reduced biomass removal (+F-R) treatment, for each region (Alb: Schwäbische Alb; Sch: Schorfheide-Chorin; Hai: Hainich-Dün), as well as for different years and seasons. Significant (< 0.05) contrasts are written in bold. Due to missing data on fertilized & biomass removal treatments in spring 2020 for the Schorfheide-Chorin no pairwise contrasts shown (*). (DOCX) [file pone.0287039.s025.docx]

**S14 Table: Pairwise comparison of biomass production across treatments.** Pairwise comparisons of biomass production in the fertilization & biomass removal (+F+R), unfertilized & reduced biomass removal (-F-R), unfertilized & biomass removal (-F+R) and fertilized & reduced biomass removal (+F-R) treatment, for each region (Alb: Schwäbische Alb; Sch: Schorfheide-Chorin; Hai: Hainich-Dün), as well as for different years and seasons. Significant (< 0.05) contrasts are written in bold. Due to missing data on fertilized & biomass removal treatments in spring 2020 for the Schorfheide-Chorin no pairwise contrasts shown (*).

| **Season** | **Region** | **Contrast** | **Estimate** | **SE** | **95% CI** | **p value** |
| --- | --- | --- | --- | --- | --- | --- |
| Summer 2020 | Alb | +F+R vs -F-R | 226.61 | 72.00 | 141.12 | **0.02** |
|  |  | +F+R vs +F-R | 228.78 | 72.00 | 141.12 | **0.02** |
|  |  | +F+R vs -F+R | -11.22 | 72.00 | 141.12 | 1.00 |
|  |  | -F-R vs +F-R | 2.17 | 72.00 | 141.12 | 1.00 |
|  |  | -F-R vs -F+R | -237.83 | 72.00 | 141.12 | **0.01** |
|  |  | +F-R vs -F+R | -240.00 | 72.00 | 141.12 | **0.01** |
|  | Hai | +F+R vs -F-R | 224.83 | 78.80 | 154.45 | **0.04** |
|  |  | +F+R vs +F-R | 217.01 | 78.80 | 154.45 | **0.05** |
|  |  | +F+R vs -F+R | -6.03 | 84.60 | 165.82 | 1.00 |
|  |  | -F-R vs +F-R | -7.82 | 78.80 | 154.45 | 1.00 |
|  |  | -F-R vs -F+R | -230.86 | 84.60 | 165.82 | **0.05** |
|  |  | +F-R vs -F+R | -223.04 | 84.60 | 165.82 | 0.06 |
|  | Sch | +F+R vs -F-R | nonEst* | NA | NA | NA |
|  |  | +F+R vs +F-R | nonEst* | NA | NA | NA |
|  |  | +F+R vs -F+R | nonEst* | NA | NA | NA |
|  |  | -F-R vs +F-R | -12.62 | 97.10 | 190.32 | 1.00 |
|  |  | -F-R vs -F+R | 191.55 | 101.80 | 199.53 | 0.26 |
|  |  | +F-R vs -F+R | 204.18 | 97.10 | 190.32 | 0.17 |
| Summer 2021 | Alb | +F+R vs -F-R | 295.47 | 64.70 | 126.81 | 0.00 |
|  |  | +F+R vs +F-R | 326.95 | 64.70 | 126.81 | 0.00 |
|  |  | +F+R vs -F+R | 5.07 | 64.70 | 126.81 | 1.00 |
|  |  | -F-R vs +F-R | 31.48 | 64.70 | 126.81 | 0.96 |
|  |  | -F-R vs -F+R | -290.40 | 64.70 | 126.81 | **< 0.001** |
|  |  | +F-R vs -F+R | -321.88 | 64.70 | 126.81 | **< 0.001** |
|  | Hai | +F+R vs -F-R | 185.07 | 64.70 | 126.81 | **0.03** |
|  |  | +F+R vs +F-R | 193.76 | 64.70 | 126.81 | **0.02** |
|  |  | +F+R vs -F+R | 32.93 | 64.70 | 126.81 | 0.96 |
|  |  | -F-R vs +F-R | 8.68 | 64.70 | 126.81 | 1.00 |
|  |  | -F-R vs -F+R | -152.14 | 64.70 | 126.81 | 0.10 |
|  |  | +F-R vs -F+R | -160.83 | 64.70 | 126.81 | 0.08 |
|  | Sch | +F+R vs -F-R | 187.14 | 79.20 | 155.23 | 0.10 |
|  |  | +F+R vs +F-R | 87.27 | 79.20 | 155.23 | 0.69 |
|  |  | +F+R vs -F+R | -9.77 | 79.20 | 155.23 | 1.00 |
|  |  | -F-R vs +F-R | -99.87 | 79.20 | 155.23 | 0.59 |
|  |  | -F-R vs -F+R | -196.91 | 79.20 | 155.23 | 0.08 |
|  |  | +F-R vs -F+R | -97.04 | 79.20 | 155.23 | 0.62 |
