# Supplementary material for: Biomass removal promotes plant diversity after short-term de-intensification of managed grasslands
Source: PLoS One. 2023 Jun 29;18(6):e0287039. doi: 10.1371/journal.pone.0287039 (PMC10310043; doi:10.1371/journal.pone.0287039)
Supplement: S15 Table — (DOCX) [file pone.0287039.s026.docx]

**S15 Table: Pairwise comparison (PERMANOVA) of plant community composition per treatment in 2020** between the fertilized & biomass removal (+F+R), unfertilized & reduced biomass removal (-F-R), fertilized & reduced biomass removal (+F-R), unfertilized & biomass removal (-F+R) treatments for all three regions (Alb: Schwäbische Alb; Sch: Schorfheide-Chorin; Hai: Hainich-Dün) and for both spring and summer 2020 (see S9 Fig).

| **Season** | **Region** | **Pairs** | **Df** | **R2** | **p value** | **p adjusted** |
| --- | --- | --- | --- | --- | --- | --- |
| Spring | Alb | +F+R vs -F-R | 1 | 0.01 | 0.93 | 1.00 |
|  |  | +F+R vs +F-R | 1 | 0.01 | 0.89 | 1.00 |
|  |  | +F+R vs -F+R | 1 | 0.01 | 0.92 | 1.00 |
|  |  | -F-R vs +F-R | 1 | 0.03 | 0.73 | 1.00 |
|  |  | -F-R vs -F+R | 1 | 0.00 | 1.00 | 1.00 |
|  |  | +F-R vs -F+R | 1 | 0.03 | 0.70 | 1.00 |
|  | Hai | +F+R vs -F-R | 1 | 0.02 | 0.87 | 1.00 |
|  |  | +F+R vs +F-R | 1 | 0.00 | 1.00 | 1.00 |
|  |  | +F+R vs -F+R | 1 | 0.07 | 0.59 | 1.00 |
|  |  | -F-R vs +F-R | 1 | 0.02 | 0.82 | 1.00 |
|  |  | -F-R vs -F+R | 1 | 0.14 | 0.36 | 1.00 |
|  |  | +F-R vs -F+R | 1 | 0.10 | 0.52 | 1.00 |
|  | Sch | -F-R vs +F-R | 1 | 0.02 | 0.95 | 0.95 |
|  |  | -F-R vs -F+R | 1 | 0.06 | 0.85 | 0.95 |
|  |  | +F-R vs -F+R | 1 | 0.11 | 0.52 | 0.95 |
| Summer | Alb | +F+R vs -F-R | 1 | 0.03 | 0.73 | 0.93 |
|  |  | +F+R vs +F-R | 1 | 0.03 | 0.75 | 0.93 |
|  |  | +F+R vs -F+R | 1 | 0.05 | 0.61 | 0.93 |
|  |  | -F-R vs +F-R | 1 | 0.00 | 0.99 | 0.99 |
|  |  | -F-R vs -F+R | 1 | 0.03 | 0.77 | 0.93 |
|  |  | +F-R vs -F+R | 1 | 0.04 | 0.66 | 0.93 |
|  | Hai | +F+R vs -F-R | 1 | 0.02 | 0.81 | 0.96 |
|  |  | +F+R vs +F-R | 1 | 0.04 | 0.63 | 0.96 |
|  |  | +F+R vs -F+R | 1 | 0.03 | 0.71 | 0.96 |
|  |  | -F-R vs +F-R | 1 | 0.00 | 0.96 | 0.96 |
|  |  | -F-R vs -F+R | 1 | 0.02 | 0.83 | 0.96 |
|  |  | +F-R vs -F+R | 1 | 0.02 | 0.80 | 0.96 |
|  | Sch | +F+R vs -F-R | 1 | 0.04 | 0.77 | 1.00 |
|  |  | +F+R vs +F-R | 1 | 0.01 | 0.92 | 1.00 |
|  |  | +F+R vs -F+R | 1 | 0.03 | 0.88 | 1.00 |
|  |  | -F-R vs +F-R | 1 | 0.01 | 0.92 | 1.00 |
|  |  | -F-R vs -F+R | 1 | 0.00 | 1.00 | 1.00 |
|  |  | +F-R vs -F+R | 1 | 0.00 | 0.97 | 1.00 |
