# Supplementary material for: Biomass removal promotes plant diversity after short-term de-intensification of managed grasslands
Source: PLoS One. 2023 Jun 29;18(6):e0287039. doi: 10.1371/journal.pone.0287039 (PMC10310043; doi:10.1371/journal.pone.0287039)
Supplement: S16 Table — (DOCX) [file pone.0287039.s027.docx]

**S16 Table: Permutation test of fitted vectors of the environmental variables (standing biomass, light availability and soil moisture), plant species richness and Shannon diversity in 2020** on the NMDS ordination (NMDS1 and NMDS2) for spring and summer of 2020 across all regions (Schwäbische Alb, Hainich-Dün, Schorfheide-Chorin) (S10 Fig).

| **Season** | **Factor** | **NMDS1** | **NMDS2** | **R2** | **p value** |
| --- | --- | --- | --- | --- | --- |
| Spring | Standing biomass | -0.06 | -1.00 | 0.05 | 0.43 |
|  | Soil moisture | 1.00 | 0.00 | 0.01 | 0.79 |
|  | Light availability | -0.04 | 1.00 | 0.02 | 0.73 |
|  | Richness | 0.72 | -0.69 | 0.09 | 0.21 |
|  | Diversity | 0.05 | -1.00 | 0.06 | 0.35 |
| Summer | Standing biomass | 1.00 | 0.04 | 0.01 | 0.81 |
|  | Soil moisture | -0.93 | -0.36 | 0.01 | 0.80 |
|  | Light availability | 0.38 | 0.93 | 0.00 | 0.97 |
|  | Richness | -1.00 | 0.02 | 0.11 | 0.03 |
|  | Diversity | -1.00 | -0.01 | 0.04 | 0.34 |
