# Supplementary material for: Biomass removal promotes plant diversity after short-term de-intensification of managed grasslands
Source: PLoS One. 2023 Jun 29;18(6):e0287039. doi: 10.1371/journal.pone.0287039 (PMC10310043; doi:10.1371/journal.pone.0287039)
Supplement: S17 Table — (DOCX) [file pone.0287039.s028.docx]

**S17 Table:** **Mean LUI drivers** (grazing, mowing and fertilization) averaged across 2017 to 2019 for each region separately (Alb: Schwäbische Alb, Hai: Hainich-Dün, Sch: Schorfheide-Chorin).

| **Region** | **Grazing**  (Livestock units * d ha^-1^ year^-1^) | **Mowing**  (cuts year^-1^) | **Fertilization**  (kg N m^-3^ year^-1^) |
| --- | --- | --- | --- |
| Alb | 0.29 | 1.93 | 3.34 |
| Hai | 0.32 | 1.48 | 3.78 |
| Sch | 2.41 | 0.51 | 1.10 |
