# Supplementary material for: Biomass removal promotes plant diversity after short-term de-intensification of managed grasslands
Source: PLoS One. 2023 Jun 29;18(6):e0287039. doi: 10.1371/journal.pone.0287039 (PMC10310043; doi:10.1371/journal.pone.0287039)
Supplement: S18 Table — Calibration was performed for data measured in spring 2021. (DOCX) [file pone.0287039.s029.docx]

**S18 Table:** **Calibration model of biomass estimates** based on a linear regression between actual standing biomass measurements (dry matter in g m^-2^) and rising plate meter measurement (1/2 cm increments), combined for all regions. Calibration was performed for data measured in spring 2021.

|  | **Estimate** | **SE** | **95% CI** | **p value** |
| --- | --- | --- | --- | --- |
| Intercept | -38.66 | 4.70 | 9.21 | < 0.01 |
| Biomass measurement | 8.68 | 0.23 | 0.45 | < 0.01 |
