# Supplementary material for: Biomass removal promotes plant diversity after short-term de-intensification of managed grasslands
Source: PLoS One. 2023 Jun 29;18(6):e0287039. doi: 10.1371/journal.pone.0287039 (PMC10310043; doi:10.1371/journal.pone.0287039)
Supplement: Attachment — Submitted filename: Response to Reviewers.docx [file pone.0287039.s033.docx]

**Editor’s comments**

We very much appreciate that the editor finds our study interesting and for giving us a chance to resubmit the manuscript. Regarding the specific comments by the editor, we refer to our responses on the more detailed comments below.

#1. What is the difference between intensification and extensification? If different, please explain. If actually the same, then I suggest using “intensification” consistently.

We thank the editor for this useful suggestion on clarifying the difference between ‘intensification’ and ‘extensification’. As a short answer here: with land-use extensification we are referring to the opposite of land-use intensification, hence a reduction in land-use intensity (i.e. reduction in mowing frequency, grazing or fertilization intensity). We now provided a definition in the introduction to improve clarity about the terminologies used (see changes in lines 56-57).

#2. Must the experiment be justified in a BEF context? Why not simply a matter of understanding land use intensification effects on biodiversity? In other words, the BEF context may not be needed and only adds an extra layer of inference here, whereas biodiversity seems worthy of preserving regardless. Think about it.

We originally included the BEF context to stress the importance of why we need to advance our understanding on how land-use drives biodiversity loss, as this loss may have detrimental consequences for ecosystem functioning. However, as the editor correctly points out, that rather adds an additional layer of complexity to our study, while biodiversity in itself is worthy of preserving regardless. Thus, we agree to the editor’s suggestion that our introduction would benefit from refining our focus and now excluded the link to the BEF context (the sentences on lines 51-53 of our original manuscript has been removed).

#3. Shannon diversity: please define with its equation. It could be the original H’, but as Jost (2006) nicely demonstrated, that is an entropy that requires exp(H’) to obtain effective diversity. Thus “Shannon diversity” has taken on several meanings of late. I suggest calculating and using Jost’s effective diversity, which corresponds to Hill number 1 (1D).

Following this suggestion, we now adjusted all our analyses by using exp(H’) instead of just H’. We now also provided the specific formula used to calculate effective diversity in the methods section (see changes in lines: 210-212). While most of the results did not change after using the exp(H’), as the only qualitative difference we now find that indirect effects of biomass removal on diversity were not only mediated via changes in light availability, but *also* via soil moisture (Fig. 4, Table 1; lines 369-371, 489-493). Hence, using effective diversity revealed the relative importance of soil moisture in summer as an indirect pathway mediated by biomass removal, similar to what we observed for species richness, making our findings more consistent.

#4. Fig. 1 Are the numbers related to a sequence (implied by numbering)? If not, perhaps they can be removed. Also, I suggest more obviously dashed lines for negative effects: on the pdf the resolution is too low, meaning that dashes are not clearly different from the solid lines – especially when zoomed out to be comparable to the size of a figure in a printed journal page (imagine figures squeezed into a column or ½ width of paper).

Yes, the numbers within the figure are related to a sequence of supporting references for each path within the causal framework and hence, we would prefer to keep the numbers within the figure. We now clarified this issue in line 108. The specific references are included in the figure caption.

#5. Also, I suggest more obviously dashed lines for negative effects: on the pdf the resolution is too low, meaning that dashes are not clearly different from the solid lines – especially when zoomed out to be comparable to the size of a figure in a printed journal page (imagine figures squeezed into a column or ½ width of paper). MOST IMPORTANTLY: Resolution of all Figures in the received pdf (and what reviewers see) is too low, so that text in figures is illegible and shapes are very indistinct. I expect negative reviews based only on this technical problems rather than merits of the work. According to the PLOS One office, "It appears the reason this error is occurring is because the authors provided figure files that are incompatible with Editorial Manager's PDF compilation. We encourage authors to use the the PACE tool (https://pacev2.apexcovantage.com/) on all of their Fig files to ensure the images will be of the highest quality in PDF form." Thus I strongly suggest following those PLOS figure guidelines for a revised submission.

We agree, and apologize for the low figure quality of the figures we provided. We now increased the quality of all figures (and ensured journal requirements for all figures using the PACE tool) within the document and, to better visualize negative of positive hypothesised relationships, also selected a different line-type for the dashed lines.
