# Supplementary material for: Biomass removal promotes plant diversity after short-term de-intensification of managed grasslands
Source: PLoS One. 2023 Jun 29;18(6):e0287039. doi: 10.1371/journal.pone.0287039 (PMC10310043; doi:10.1371/journal.pone.0287039)
Supplement: Attachment — Submitted filename: Response to Reviewers.docx [file pone.0287039.s034.docx]

**Editor’s comments**

I find Reviewer 1 is generally complementary about the manuscript and provides good suggestions. As usual, the reviewer and I see different features to emphasize – a good thing! I especially agree with the reviewer in the suggestion that the short-term effects should be emphasized, which would be consistent with my comments below about potential, and acknowledged, lag effects. Reviewer 1 suggested Minor Revisions, but I think the aggregate of their comments and mine add up to Major Revision (for what that’s worth). I also think that having resolved or dispelled comments here, the manuscript should be ready to accept. Please see the details on Reviewer 1’s comments via the information provided by PLOS One. Below are my comments on the manuscript. Finally, thanks for your patience in this review process.

We very much appreciate the complementary comments provided by both the editor and the reviewer. We fully agree with the general recommendation to emphasize short-term and lag effects and adjusted the respective sections in our discussion (see changes in lines: 39, 126-128, 451-454, 541, 557-558). Regarding the specific comments by the editor and the reviewers, we refer to our responses to the more detailed comments below.

Title and lines 56-57 and elsewhere. The word “extensification” is not the opposite of “intensification” and is not used by the cited papers [9, 10]. If I look it up in online dictionaries I see definitions like this: 1. the process of making something (more) extensive 2. the geographic spread and distribution of any technology, especially agriculture. Thus I suggest it be replaced with an antonym, such as one of those listed at https://www.thesaurus.com/browse/intensification? Or perhaps "mitigation"? Or more clumsy: "de-intensification"?

We thank the editor for this comment and now used the term “de-intensification” when referring to reduction of land-use intensity throughout the manuscript and within our title (“Biomass removal promotes plant diversity after short-term de-intensification of managed grasslands”). See also lines 1-2, 56, 110, 127 and 154.

Line 247. I expected SEM software etc. to be listed in this section.

We agree and now added the SEM software in line 257.

It may help to re-organize subheadings?

We agree and re-organized and re-named the subheadings in the methods section to allow a better distinction between “conventional” statistical methods (linear mixed effect models and pairwise contrasts) and more in-depth analyses using multivariate analyses (NMDS) as well as piecewiseSEM. Specifically, we refined subheadings in the methods (see lines: 258, 272, 284) and result section (see lines: 324).

Line 255. I strongly encourage authors to move beyond repeated pairwise Tukey tests and rely more on GLMs and SEMs for interpreting statistical trends.

We fully agree with the editor that GLMs and SEMs provide more in-depth statistical results compared to analyses based on pairwise Tukey tests. We noticed that we communicated this wrongly in our original manuscript and apologize for the confusion:

In our original manuscript we did not use the Tukey HSD test (as incorrectly mentioned in our methods section) to compute pairwise differences. Rather, after testing for overall effects of treatments within our Linear Mixed Model, we applied the more advanced *emmeans* function from the package “emmeans”, which computes contrasts between treatments based on estimated least-square means. To clarify this information we corrected all respective description about pairwise tests throughout our manuscript (see lines: 261-263, 327-328).

In our manuscript we deliberately analysed differences between treatments using “conventional” Linear Mixed Models as a first step, to test for treatment effects. However, we also agree with the editor that more advanced statistical methods are needed to provide more insights into the mechanisms (e.g. through changes in vegetation biomass [and hence light competition], or changes in soil moisture) by which these treatments influence plant biodiversity indicators. Hence, in our manuscript we used piecewiseSEM for this purpose (methods: section 3.5.3, results: section 4.2).

Paragraph starting at line 292. I strongly recommend the authors use AIC.psem in the piecewise SEM package to compare models for “efficiency” or “plausibility.” This will provide a more robust test of which model to spend more time with than other tests, and then leaves goodness of fit measures as a way to criticize the most efficient model selected by AIC. See extensive literature by Burnham and Anderson for more details on AICs. The AIC results should then be a table in the manuscript, followed by results for the "winning" model.

We thank the editor for this useful suggestion and now compared the respective models using AIC.psem from the piecewise SEM package. Accordingly, in the methods we now explained that we compared the AICs of each model when alternative model structures were tested (see lines: 304-311, 375-379).

Specifically, we started with constructing *a priori* models (separately for richness and diversity in spring and summer) based on our hypothetical framework introduced in our paper. If alternative pathways improved model fit, we now additionally compared competing models based on the AIC. This was only done in summer, as there, an error correlation between biomass production and soil moisture improved overall model fit. Hence, we reported these AIC results in our manuscript. However, as model fit of our *a priori* developed spring models was already good, we did not compare any competing models with the spring models, in line with the philosophy that ideally, only *a priori* developed models in SEMs are shown, unless model fit suggests that initial models are not adequate yet (see lines: 306-311).

Fig. 2 revealed little to me. I recommend the authors instead show and discuss a table of GLM output from lme4: are coefficients for biomass removal and fertilizer addition clearly non-zero? What are the signs? What is the overall fit?

We agree, and now put Figure 2 of the original manuscript into our supplement, and additionally provided a table showing the standardized effect sizes of the treatment effects on species richness in the main manuscript (see Table 1, lines 335-341). In line with the suggestions from the editor, this table now more clearly shows the sign, confidence intervals and overall fit for each model.

Speaking of regression output, S1 Table is close to what I sought, but I think it reveals errors that matter:
• Why is is Spring 2020 different from other time steps in its model structure?

The statistical output for the model testing the treatment effect on species richness in spring 2020 is different compared to the other seasons or years, as the most parsimonious model (based on AIC) did not contain an interaction between treatment and the respective regions. To emphasise this fact, we now provided some additional information in the table caption (see lines in supplement: 113-114).

• The intercept (default by R) is Alb but also +F + R. It may make more sense to choose (using relevel in R) to use Alb but -F -R for a control condition as the intercept.

While we agree that from a theoretical point of view, -F-R might be a more reasonable default, we deliberately choose +F+R as default, as in our experimental design the “unmanipulated” plots were +F+R (where land-use intensity reflected the local management by the respective farmer). Hence, we would prefer to keep +F+R as default in our models, unless the editor wishes otherwise.

Also I suggest converting SE to 95% confidence intervals (1.96 X SE) and omitting the DF and t value columns. And note that rows are relative to the intercept.

We agree and now provided the 95% CI in all our tables, omitted the DF and t-values, and added the note that rows are relative to the intercept.

Finally, the study was designed as if site and time could be random effects, though with too few levels of each to really help as random effects. That makes all the sites and times show as separate estimates. The minimum number of levels needed for random effects in a mixed effect model may not be met here (that threshold is not clear), but I suggest trying a GLMM instead of the GLM. That would potentially move sites and time into random effects, leaving main effects of interest as fixed effects and making interpretation more straight-forward. I may be wrong - it may not work because sample size is limiting. If so, please just say so in your response to review comments. But - if it works, it may be more efficient and effective as a test of treatment effects.

We fully agree that a GLMM with regions and year as random effects would help to correct our estimates. However, unfortunately, our study design lacks the required number of levels (region only 3, year only 2) for these variables to be used as random effects (rough threshold of minimal 5 levels, see Chapter 11, Section 5 in “Data Analysis Using Regression and Mulitilevel/Hierarchical models” by Gelman and Hill, 2007). Hence, while appreciating the suggestion by the editor, a GLMM with random effects would unfortunately not improve our current modelling approach.

Line 313. This sentence could be edited to be more direct.

We agree, and reformulated and restructured this sentence to be more direct (see line: 326-329).

Beyond that, this section 4.1 based on regression could be argued (above?) as seeking overall (i.e., direct and indirect) effects of treatments on diversity, whereas SEM seeks to tease apart the direct from the indirect. Thus regression evaluates overall / net effects while SEM evaluates details. Perhaps that can help reconcile the greater information in Fig. 4 than in Fig. 2. And by the way, the use of SEM is an advance to make clear for this research subject – much research has not used it but work here shows it helps bring clarity.

We fully agree and appreciate the editors recognition of our use on more “advanced” statistical methods, such as SEM, in addition to “conventional” statistics, to distinguish between direct and indirect treatment effects. According to the editors suggestions, we now renamed subheadings in the methods section and in the results section, to clarify how the different statistical approaches used in our study answer different questions. We hope that this will also help the reader to reconcile the greater information in Fig. 2.

Line 417. I don’t know what is meant by “land use reductions.”

We reformulated the sentence to increase clarity (see line: 431-433).

Paragraph starting at Line 425 and subsequent paragraphs. It is usually expected that Figures are not cited in Discussion, to ensure that Results are clearly separate from Discussion. If it feels like Figures need to be used, then the sentence may belong better in Results.

We agree with the suggestion of the editor and removed all references to Figures or Tables from the discussion.

Line 438. Yes – I agree; time lags are often observed, and some citations to support this would be useful here.

We agree and now provided additional references on lag effects found in previous studies (see line: 454).

Paragraph starting at Line 448. This paragraph covers almost 3 pages! Organize and subdivide.

We apologize for the inconvenient format of this paragraph and re-organized the respective section into three separate paragraphs (see re-organized paragraphs in lines: 464-538).

Line 522. Careful! Not only is “extensification” used here again, but I think the authors need to be careful to avoid claiming a first. Consult the rich literature by Tilman and a small army of others at Cedar Creek. In addition, other studies that used a broader range of fertilizer and biomass removal have explored hump-shaped patterns related to the Intermediate Disturbance Hypothesis, Intermediate Productivity Hypothesis, and Huston’s combination of those two ideas (his Dynamic Equilibrium Model). There is a long history of inquiry here that includes factors here as part of the story, as the co-authors well know (they have been involved in some). Among others, a few that may be relevant include:
• Feng-Wei Xu, Jian-Jun Li, Li-Ji Wu, Xiao-Ming Lu, Wen Xing, Di-Ma Chen, Biao Zhu, Shao-Peng Wang, Lin Jiang, Yong-Fei Bai, Resource enrichment combined with biomass removal maintains plant diversity and community stability in a long-term grazed grassland, Journal of Plant Ecology, 113(5), October 2020, Pages 611–620, https://doi.org/10.1093/jpe/rtaa046
• Band, N., Kadmon, R., Mandel, M. and DeMalach, N., 2022. Assessing the roles of nitrogen, biomass, and niche dimensionality as drivers of species loss in grassland communities. Proceedings of the National Academy of Sciences, 119(10), p.e2112010119.
• Carnus, T., Connolly, J., Kirwan, L. and Finn, J.A., 2013. Plant diversity effects are robust to cutting severity and nitrogen application in productive grasslands. In The role of grasslands in a green future: threats and perspectives in less favoured areas. Proceedings of the 17th Symposium of the European Grassland Federation, Akureyri, Iceland, 23-26 June 2013 (pp. 195-197). Agricultural University of Iceland.

We thank the editor for this important comment and apologize for the confusion. We fully agree, that there are many previous studies, investigating the effect of different land-use drivers on plant biodiversity. To clarify this, we now rephrased the respective sections in our discussion (see lines: 539-543), emphasizing that we build on knowledge of previous studies. Additionally, we now specifically refer to our experimental design (high land-use intensity as a default in comparison to treatments in which the intensity of certain land-use drivers was reduced) to emphasize the novelty of our study compared to previous research (see lines: 541-543). Finally, we very much appreciated the useful references provided and referred to them in our manuscript.

I think the concluding paragraph could benefit from a sentence or two on limits of the study – as difficult as it is to get this much data, it still has limits in the number places and times, for strong analyses and to capture acknowledged potential lag effects.

We fully agree, and now provided some additional information on the limitations of our study in the discussion section and highlighted avenues for future research (see lines: 557-558, 560-563).

**Reviewer #1:**

Reviewer #1: The authors present results on plant biodiversity, community composition and plant biomass from an in-field experiment in which the land-use components fertilization and biomass removal have been reduced in a full-factorial design. The results represent the short-term effects one and two years after the experiments have been implemented. Although effects on the plant community are very small overall, the study does provide interesting insights into possible mechanistic pathways as the authors also included light availability and soil moisture in their models. Both the experimental design and the statistical analyses are done rigorously and correctly, hence the conclusions are supported by the data.

We thank the reviewer for the positive feedback on our study. Regarding the specific comments by the reviewer, we refer to our responses to the more detailed comments below.

* The main strength of the experiment compared to previous studies is the implementation of all treatments within the same grassland sites. This could be emphasised more in the introduction.

We thank the reviewer for this suggestion and now emphasized our experimental treatment within the introduction (see lines: 102-105).

* The introduction lacks an explanation of the expected "seasonal variability of relationships" mentioned in the legend of Figure 1. As spring and summer data are analysed and discussed separately, a section on seasonal differences should be added in the introduction.

We agree and now added two sections providing further rational on why we expect “seasonal variability of relationships” in our introduction (see lines: 66-69, 88-90).

* The (indirect) effect of biomass production on diversity is mentioned in the hypotheses, but not explained and motivated in the Introduction. As biomass production is an important component in the model structure, the expected effects and mechanisms should be added to the introduction.

We now clarified our motivation why indirect effects of fertilization are mediated by changes in biomass production in our introduction (see lines: 63-66).

* The experiments were set up in grasslands of different land-use intensity. While the average intensity values are given in the Supplement, the description in the Methods section is inconsistent: In line 142f "a gradient in land-use intensity" is mentioned, while in line 163 management is described as "relatively high" and in line 163 as "medium to high". In addition to making the statements consistent, I recommend describing the range of land-use components on the grasslands by the actual number and time of cuts, amount of fertilizer and/or number of grazing animals (and not just in terms of the standardized land-use intensity).

We apologize for the inconsistency when describing the land-use intensity gradient at the given grasslands and now provided information on the range of the intensity of each land-use driver in our methods section (see lines: 171-173).

* The description of the treatments is in some parts difficult to read, especially if several treatments are mentioned in one sentence. I would prefer the use of abbreviations as done for the supplementary tables. The authors could also consider calling the treatment with fertilizer and biomass removal the control.

We appreciate the useful suggestion to use the treatment abbreviations and adjusted the treatment names throughout the manuscript. Within the figures, we only provided the full treatment names, while in the caption, we referenced both the full and the abbreviated treatment name. This was done to guarantee the connection between abbreviated and full treatment names, to make sure readers understand our figures even if they did not read the full manuscript.

* The terms subplot, plot and field are used inconsistently in the first part of the manuscript. See for instance line 176f. Please use consistent terminology and I would recommend not using "field" but rather "grassland".

We fully agree and now clarified terminologies throughout the manuscript when referring to “plots” or “subplots”. Additionally, we now use “grassland” instead of “field” throughout the manuscript.

**Minor comments:**

Line 146: "the 10 most abundant"

We agree and added quotation marks in line 156.

Line 171: Why was the fertilizer manually applied in this treatment? Why was is not fertilized together with the "control" treatment and the remaining grassland?

The +F-R and the -F-R subplots were both located in a plot where land-use was reduced. While in the +F+R, the farmer applied fertilizer using heavy machinery (fertilizer is sprayed over relatively large area), fertilizer was manually applied in the +F-R subplots, to avoid cross-contamination between treatments. We clarified this information in the methods section, see lines: 179-182.

Line 173f: This information can be moved to the next section on field data collection

We agree and deleted the respective text from the “Experimental design” section.

Line 158: What does "setting-up" mean exactly? Does this mean that the plots were marked or was e.g. fertilization in autumn 2019 already reduced? Please clarify in which year/season the treatments were applied for the first time.

With “setting-up” we refer to plots, that were marked and, in some cases (+F-R and -F-R) fenced in autumn 2019. Reduction in both biomass removal or fertilization was started from autumn 2019 onwards. We specified this information and provided further clarification in our method section (see lines: 167-168).

Line 243 ff: This sentence contains the same information twice.

We now rephrased the respective sentence (see lines: 248-251).

Line 254: remove "with"

We adjusted respective sentence.

Line 257: which additional predictors are those?

To test how species richness differed between treatments, we stepwise added “treatment” and “region”. The predictor “treatment” was always kept in the final model, irrespective of whether it was part of the most parsimonious model or not. We clarified this information in the methods section in line: 264-267, 269-271.

Line 313 ff: there is no need to mention the type of treatment again in the parentheses#

We agree and adjusted the respective sentences.

Line 350f (and later): it is not quite clear what the "respectively" refers to. The path coefficients should be given directly after richness and diversity.

We agree and adjusted the respective sentences (see line: 362-364).

Figure 2: the y-axis label seems incorrect (remove the minus)

Species richness was quantified as the number of vascular plant species within a 1x1 m subplot. Hence, we used the label “species richness (m^-2^)” to indicated that species richness is given *per* square meter.
